# Cytomegalovirus inhibitors of programmed cell death restrict antigen cross-presentation in the priming of antiviral CD8 T cells

**DOI:** 10.1371/journal.ppat.1012173

**Published:** 2024-08-15

**Authors:** Stefan Ebert, Verena Böhm, Julia K. Büttner, Wolfram Brune, Melanie M. Brinkmann, Rafaela Holtappels, Matthias J. Reddehase, Niels A. W. Lemmermann

**Affiliations:** 1 Institute for Virology, University Medical Center of the Johannes Gutenberg-University Mainz, Mainz, Germany; 2 Leibniz Institute of Virology (LIV), Hamburg, Germany; 3 Institute of Genetics, Technische Universität Braunschweig, Braunschweig, Germany; 4 Virology and Innate Immunity Research Group, Helmholtz Centre for Infection Research, Braunschweig, Germany; 5 Research Center for Immunotherapy (FZI), University Medical Center of the Johannes Gutenberg-University Mainz, Mainz, Germany; 6 Institute of Virology, Medical Faculty, University of Bonn, Bonn, Germany; University of Zurich, SWITZERLAND

## Abstract

CD8 T cells are the predominant effector cells of adaptive immunity in preventing cytomegalovirus (CMV) multiple-organ disease caused by cytopathogenic tissue infection. The mechanism by which CMV-specific, naïve CD8 T cells become primed and clonally expand is of fundamental importance for our understanding of CMV immune control. For CD8 T-cell priming, two pathways have been identified: direct antigen presentation by infected professional antigen-presenting cells (pAPCs) and antigen cross-presentation by uninfected pAPCs that take up antigenic material derived from infected tissue cells. Studies in mouse models using murine CMV (mCMV) and precluding either pathway genetically or experimentally have shown that, in principle, both pathways can congruently generate the mouse MHC/H-2 class-I-determined epitope-specificity repertoire of the CD8 T-cell response. Recent studies, however, have shown that direct antigen presentation is the canonical pathway when both are accessible. This raised the question of why antigen cross-presentation is ineffective even under conditions of high virus replication thought to provide high amounts of antigenic material for feeding cross-presenting pAPCs. As delivery of antigenic material for cross-presentation is associated with programmed cell death, and as CMVs encode inhibitors of different cell death pathways, we pursued the idea that these inhibitors restrict antigen delivery and thus CD8 T-cell priming by cross-presentation. To test this hypothesis, we compared the CD8 T-cell responses to recombinant mCMVs lacking expression of the apoptosis-inhibiting protein M36 or the necroptosis-inhibiting protein M45 with responses to wild-type mCMV and revertant viruses expressing the respective cell death inhibitors. The data reveal that increased programmed cell death improves CD8 T-cell priming in mice capable of antigen cross-presentation but not in a mutant mouse strain unable to cross-present. These findings strongly support the conclusion that CMV cell death inhibitors restrict the priming of CD8 T cells by antigen cross-presentation.

## Author summary

In patients as well as in experimental mouse models, CD8 T cells represent the most potent antiviral effector cells in preventing CMV disease in immunocompromised recipients of hematopoietic cell transplantation. Despite the clinical relevance of mounting a protective response, the mode of CMV antigen presentation to naïve CD8 T cells was long debated. In principle, naïve CD8 T cells can be sensitized through “direct antigen presentation” on the surface of infected professional antigen-presenting cells (pAPCs) or through “antigen cross-presentation" by uninfected pAPCs that take up antigenic material derived from infected cells following cell death. As CMVs are cytopathogenic, eventually killing host cells, and as they encode immune evasion proteins interfering with the MHC/HLA class-I pathway of direct antigen presentation, it seemed reasonable to propose a dominant role for antigen cross-presentation. Mouse models precluding either mode of antigen presentation, however, revealed that both can raise an equivalent CD8 T-cell response, and recent work has identified direct antigen presentation as the canonical pathway taken when both are accessible. Here we show that virus-encoded inhibitors of two programmed cell death modalities, apoptosis and necroptosis, reduce antigen release to below a level required for efficient cross-presentation. This solves an open question in CMV immunology.

## Introduction

Medical interest in cytomegaloviruses (CMVs), members of the β-subfamily of the herpes virus family [1], is based on life-threatening disease that human cytomegalovirus (hCMV) causes in fetuses, newborn infants, and immunocompromised patients [2–4]. Risk groups in transplantation centers worldwide are recipients of hematopoietic cell transplantation (HCT) [5] and solid organ transplantation (SOT) [6], making hCMV a medically relevant pathogen with an impact on individual health and public health economy [7,8].

CMVs are host species-specific [9–11] as a result of eons of co-evolution with and adaptation to their specific mammalian hosts, leading to the acquisition of “private genes” not shared between different CMV species [1,12]. As far as analyzed, most of these viral private genes are involved in the interaction between the virus and the host’s immune system. As revealed by the Bayesian evolutionary tree for the genus *Cytomegalovirus* [1,13,14], CMV species have segregated molecularly from a common ancestor and retained homologous genes involved in the viral replication machinery and in the interaction with cellular signaling pathways conserved between mammalian host species. Examples include viral inhibitors of programmed cell death [15–17]. Importantly, private viral genes often have analogous functions reflecting the evolutionary principle of “biological convergence” [13]. For instance, hCMV and non-human CMVs, such as murine CMV (mCMV), have independently evolved non-homologous genes interfering by quite different molecular mechanisms with the MHC/HLA class-I (MHC-I) pathway of direct antigen presentation to CD8 T cells [18–20].

Based on homologous as well as functionally analogous genes, key features of hCMV pathogenesis and immune control can be reproduced in animal CMV-host pairs. These include the facts that (1) productive primary infection is rapidly cleared in the immunocompetent host by effector mechanisms of innate and adaptive immunity without overt clinical symptoms [18], (2) viral genomes are not eliminated in the course of termination of the productive infection but are maintained in certain cell types in a latent state defined by absence of coordinated productive cycle gene expression, and can be reactivated to productive infection [21,22], and (3) primary infection of an immunocompromised host is productive and cytolytic, leading to intra-tissue virus spread with extensive tissue destruction that eventually can cause multiple organ failure with lethal outcome [4,23,24].

On this basis, animal models can serve to make predictions regarding CMV disease, natural immune control, and therapeutic immune intervention in all questions that cannot be addressed by clinical investigation. Examples are experimental studies on the role of host and viral genes by using specifically tailored transgenic host mutant strains and recombinant viruses with mutations in genes of interest. Non-human primate models are closest to hCMV infection [25], but the mouse model based on infection with mCMV is the most versatile, in particular with regard to altering host genetics [26], and is best understood in terms of mechanisms [27]. The predictive value of the mouse model was revealed by the finding of the protective function of CD8 T cells in preventing cytopathogenic viral intra-tissue spread and histopathology [28–32]. Specifically, a timely reconstitution of high-avidity CD8 T cells was found to be crucial for preventing CMV disease in iatrogenically immunocompromised recipients of experimental [33–36] as well as of clinical HCT [37,38].

Given the importance of CD8 T cells, it may come as a surprise that the mode by which virus-specific but antigen inexperienced, naïve CD8 T cells are primed and clonally expand to mount a protective response has long been debated. In principle, naïve CD8 can be primed by “direct antigen presentation” [39,40], that is, recognition by their T-cell receptors (TCR) of peptide-loaded MHC-I (pMHC-I) complexes on the surface of infected professional antigen presenting cells (pAPCs), such as macrophages and dendritic cells (DCs). Alternatively, priming can be through “antigen cross-presentation” [41,42] by uninfected pAPCs that engulf and process antigenic material derived from infected cells of any cell type following cell death, such as apoptotic bodies or necroptotic extracellular vesicles [43,44]. In the end, this also leads to the recognition of cell surface pMHC-I complexes sensitizing naïve CD8 T cells.

Inspired by the finding that all CMVs encode immune evasion proteins that interfere with the cell surface expression of pMHC-I complexes [19,20], thereby inhibiting direct antigen presentation, several reports provided evidence for cross-presentation by uninfected pAPCs [45–50]. Most convincingly in our view, Snyder and colleagues [47] showed that priming by MHC-I-deficient cells infected with a spread-deficient mCMV mutant, thus excluding first and subsequent rounds of direct antigen presentation, primed the full viral epitope-specificity repertoire and epitope hierarchy known for infection of C57BL/6 (MHC/H-2^b^ haplotype) mice. Notably, we recently found essentially the same CD8 T-cell response in the mutant mouse strain C57BL/6-Unc93b1^3d/3d^ (briefly Unc93b1^3d/3d^) [51] that is known to lack endosomal TLR3, 7, and 9 signaling and is impaired in exogenous antigen processing and thus in antigen cross-presentation [52]. As conclusion, the CD8 T-cell response can be primed alternatively but congruently by either mode of antigen presentation when the respective other pathway is made unaccessible experimentally or genetically.

In two recent reports [51,53], we addressed the question of which of the two priming paths is the natural default path when both are accessible. By modulation of direct antigen presentation using recombinant viruses in which the key immune evasion gene m152 of mCMV is either deleted or overexpressed, we showed that the magnitude of the CD8 T-cell response in a draining regional lymph node (RLN) of immunocompetent mice [51] as well as during immune reconstitution after experimental HCT [53] is primarily determined by direct antigen presentation.

**Fig 1:**
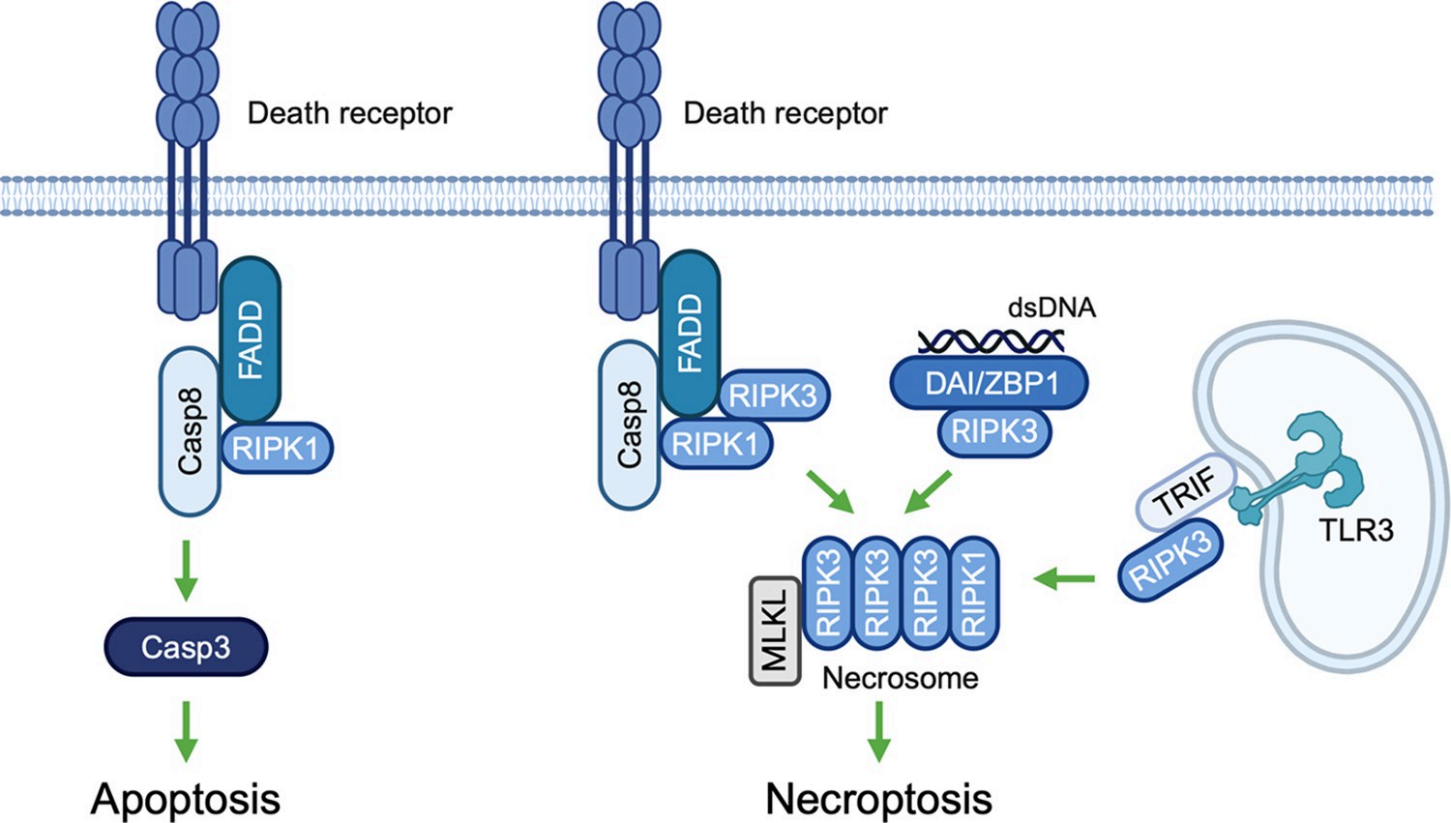
Signaling pathways of programmed cell death: brief introductory overview. (Casp3;8) caspase 3;8, (DAI/ZBP1) DNA-dependent activator of interferon-regulatory factors/Z-DNA-binding protein 1, (FADD) Fas-associated death domain protein, (MLKL) Mixed lineage kinase domain-like protein, (RIPK 1;3) receptor interacting protein kinase 1;3, (TLR3) Toll-like receptor 3, (TRIF) TIR [Toll/interleukin-1 receptor] domain-containing adaptor protein inducing interferon beta. Only the extrinsic signaling pathway is shown for apoptosis. The sketch was created with BioRender.com.

This leaves us with the unsolved question of why antigen cross-presentation, although possible in principle, does not make a notable contribution to CD8 T-cell priming after mCMV infection. Priming by cross-presentation requires the feeding of cross-presenting pAPCs, such as CD8^+^CD11c^+^ DCs [54–56], with antigenic material released from infected cells following programmed cell death, such as apoptosis and necroptosis [57–60]. Apoptosis may result from extrinsic signaling initiated by ligation of death receptors and activation of caspase-8 or from intrinsic signaling involving the activation of caspase-9, both leading to effector caspases 3, 6, and 7 [57]. Necroptosis results either from extrinsic death receptor signaling or from intrinsic signaling initiated by double-stranded (ds) nucleic acid, specifically by dsZ-RNA in the case of mCMV [61], both leading to the activation of the receptor-interacting protein kinase 3 (RIPK3) and the mixed lineage kinase domain-like (MLKL) protein (for a simplified overview, see Fig 1).

As CMVs express inhibitors of programmed cell death [17,62], we studied the CD8 T-cell response in an RLN of mice infected with recombinant mCMVs lacking either the viral inhibitor of caspase-8 activation (vICA) M36, which inhibits apoptosis [63–66], or the viral inhibitor of RIPK3-signaling M45, which inhibits necroptosis [65,67,68]. Our results confirm a dominating basal contribution of direct antigen presentation to the antiviral CD8 T-cell response, but also reveal an enhanced response to viruses in which inhibition of apoptosis or necroptosis is relieved by deletion of M36 and M45, respectively. This additive contribution to the CD8 T-cell response on top of the response to direct antigen presentation is definitely due to antigen cross-presentation, because it is missing in mice that are genetically deficient in antigen cross-presentation.

In conclusion, in normal infection, the viral inhibitors of programmed cell death, namely M36 and M45, prevent a notable contribution of antigen cross-presentation to the antiviral CD8 T-cell response.

## Results

### Deletion of the viral apoptosis inhibitor M36 leads to enhanced CD8 T-cell priming

**Fig 2:**
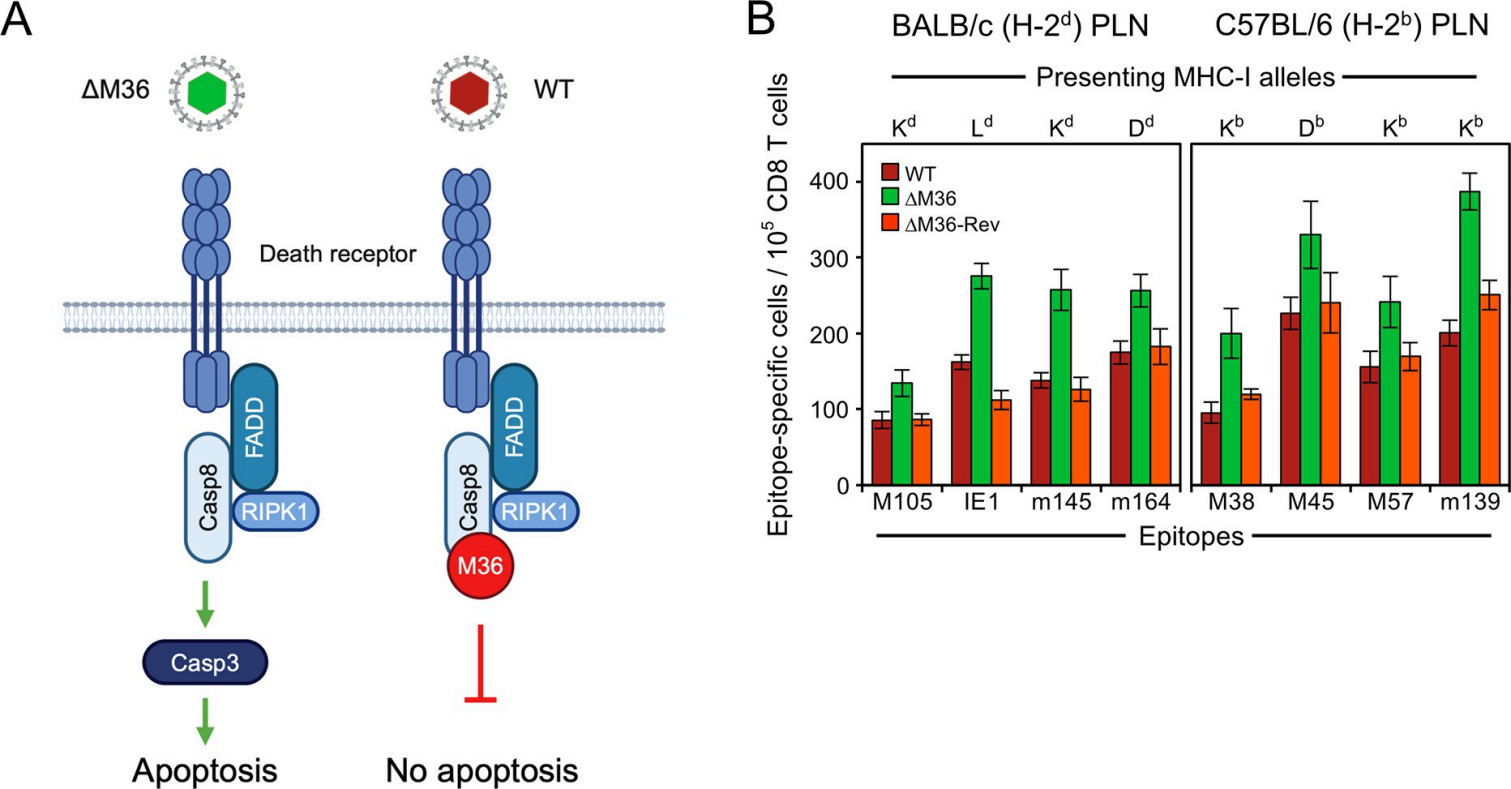
Release from apoptosis blockade by deletion of M36 enhances the antiviral CD8 T-cell response. (**A**) Sketch illustrating the block in the apoptotic signaling pathway by mCMV protein M36. (ΔM36) mCMV-ΔM36; virus symbol colored green to indicate the approval of apoptosis by deletion of M36. (WT) mCMV-WT; virus symbol colored red to indicate the inhibition of apoptosis by M36. The sketch was created with BioRender.com. (**B**) Viral epitope-specific CD8 T-cell response determined for the RLN, specifically the PLN, on day 7 after intra-plantar infection of BALB/c or C57BL/6 mice with viruses mCMV-WT (WT) expressing M36, mCMV-ΔM36 (ΔM36) lacking M36, and the revertant virus mCMV-ΔM36-Rev (ΔM36-Rev) expressing M36. (Green color) apoptosis can take place. (Red colors) apoptosis is blocked. Bars represent frequencies of responding CD8 T cells measured in ELISpot assays performed for cohorts of 5 mice per infecting virus. Error bars indicate 95% confidence intervals (CI) determined by intercept-free linear regression analysis based on graded numbers of responder cells. Differences are considered significant when the 95% CI do not overlap.

Viral inhibitor of caspase-8 activation (vICA) M36 of mCMV is the homolog of hCMV vICA UL36 [63,66,69]. Both prevent apoptosis by inhibiting caspase-8 activation (for a scheme, see Fig 2A). Interestingly, UL36 can replace M36 in its anti-apoptotic function through binding to murine pro-caspase 8, as shown by Chaudhry et al. [70] using chimeric mCMV^UL36^ expressing UL36 in place of M36.

A possible impact of UL36 on the priming of a human CD8 T-cell response cannot be studied experimentally with a UL36 deletion mutant of hCMV. We therefore employed the mouse model to study the viral epitope-specific primary CD8 T-cell response in a draining RLN, which is the popliteal lymph node (PLN) in the specific case of intra-plantar infection.

We focused on the events in the PLN because the intra-plantar route of infection is particularly well-characterized also beyond mCMV [71–75], including studies on M36 showing its role in allowing the virus to spread from the PLN to distant host tissues [76]. It is important to recall that RLNs are the site of CD8 T-cell priming also after other routes of infection (for a review, see [77]), such as the draining tracheal lymph nodes after airborne, intratracheal infection [78] or the mandibular, deep-cervical, and mediastinal lymph nodes after intranasal infection [79]. We defined day 7 as the most appropriate time point for quantification of the primary CD8 T-cell response, because productive infection is rapidly cleared in the PLN of immunocompetent mice [72], so priming of naïve CD8 T cells is essentially complete by day 7. After that time, export of primed CD8 T cells from the PLN via the efferent lymph confounds quantification of the primary response at downstream lymphoid sites, and infiltration of non-lymphoid organ sites of infection, such as the lungs, depends on recruitment of already primed cells [80].

The anti-apoptotic function of M36 was originally described to operate in infected macrophages [64,81,82], and macrophages can serve as pAPCs for the priming of a CD8 T-cell response in the PLN after intra-plantar infection with mCMV (S1 Fig). More recently, M36 was found to prevent apoptosis also in dendritic cells (DC) [83], the canonical pAPCs.

We compared the CD8 T-cell responses to wild-type (WT) mCMV (mCMV-WT), the M36 gene deletion mutant mCMV-ΔM36, and the corresponding revertant virus mCMV-ΔM36-Rev in the PLN of BALB/c (MHC haplotype H-2^d^) mice on day 7 after intra-plantar infection (Fig 2B, left panel). It must be noted that the response magnitude on day 7 reflects the initial event of the sensitization of naïve CD8 T cells as well as a few rounds of subsequent clonal expansion. Essentially all responding CD8 T cells are lymphoblasts showing the cell surface phenotype KLRG1^+^CD62L^-^ that indicates recent sensitization by antigens [84]. For four representative epitopes of mCMV presented as antigenic peptides by MHC-I molecules K^d^ (epitopes M105 and m145), L^d^ (epitope IE1), and D^d^ (epitope m164), the response to infection with mCMV-ΔM36 was consistently higher than the response to WT virus. This enhanced response was functionally reverted to WT virus level after infection with the M36-restored virus mCMV-ΔM36-Rev.

In an independent second experiment, the CD8 T-cell responses in the PLN to mutant and revertant virus were compared in the time course after intra-plantar infection of BALB/c mice (S2A Fig). The data essentially reproduced and thus confirmed the results shown for day 7 (Fig 2B, left panel) and added the information that minor differences in the initial priming event gain statistical significance over time of clonal expansion, that is, after a few rounds of proliferation [72]. Primed CD8 T cells are exported from the PLN with the efferent lymph and accumulate in the spleen. As previously shown, apoptosis of infected cells in the PLN reduces the spread of mCMV-ΔM36 to downstream organ sites of infection [76] and thus limits not only acute but also latent infection of the spleen [22]. As a consequence, CD8 T cells primed in the PLN in the course of infection with mCMV-ΔM36 do not become re-stimulated in the spleen, so that the early advantage of priming by mCMV-ΔM36 is lost over time until CD8 T cells primed and re-stimulated by mCMV-ΔM36-Rev dominate the virus-specific memory CD8 T-cell pool (S2B Fig). In detail, a high primary response in the spleen is followed by a “contraction phase” characterized by a decline in the frequencies of viral epitope-specific CD8 T cells, before frequencies for certain epitopes, specifically IE1 and m164 in BALB/c mice [85], rise again. This phenomenon is known as “memory inflation” (MI) and depends on direct presentation by non-hematopoietic cells [86–89] of antigenic peptides expressed sporadically during viral latency [90]. Accordingly, MI occurred in the spleen of mice latently infected with mCMV-ΔM36-Rev but was absent in mice infected with mCMV-ΔM36 that cannot efficiently establish a latent infection of the spleen (S2B Fig).

BALB/c is known as a mouse strain susceptible to mCMV infection, whereas C57BL/6 (MHC haplotype H-2^b^ mice) are resistant based on a strong innate immune response conducted by Ly49H^+^ natural killer (NK) cells activated by binding to the virally encoded ligand m157 (for a review article comparing the two mouse strains, see [91]).

This made it important to reproduce the findings in an analogous experimental setting by intra-plantar infection of C57BL/6 mice (Fig 2B, right panel). For four representative epitopes of mCMV presented as antigenic peptides by MHC-I molecules K^b^ (epitopes M38, M57, and m139), and D^b^ (epitope M45), the response to infection with mCMV-ΔM36 was consistently higher in the PLN than the response to WT virus. Again, this enhanced response was functionally reverted to WT virus level after infection with mCMV-ΔM36-Rev.

In conclusion from this set of data, apoptosis of infected cells enabled by deletion of M36 enhances the CD8 T-cell response in an RLN for all viral epitopes tested for two commonly used mouse inbred strains, independent of the MHC-I molecule that presents the respective antigenic peptide and unaffected by differences in the non-MHC genetic background. We thus have identified enhanced CD8 T-cell priming after deletion of M36 as a more general principle.

### Virus growth attenuation by deletion of M36 is not caused by enhanced immune control but reflects premature loss of virus-producing cells due to apoptosis

**Fig 3:**
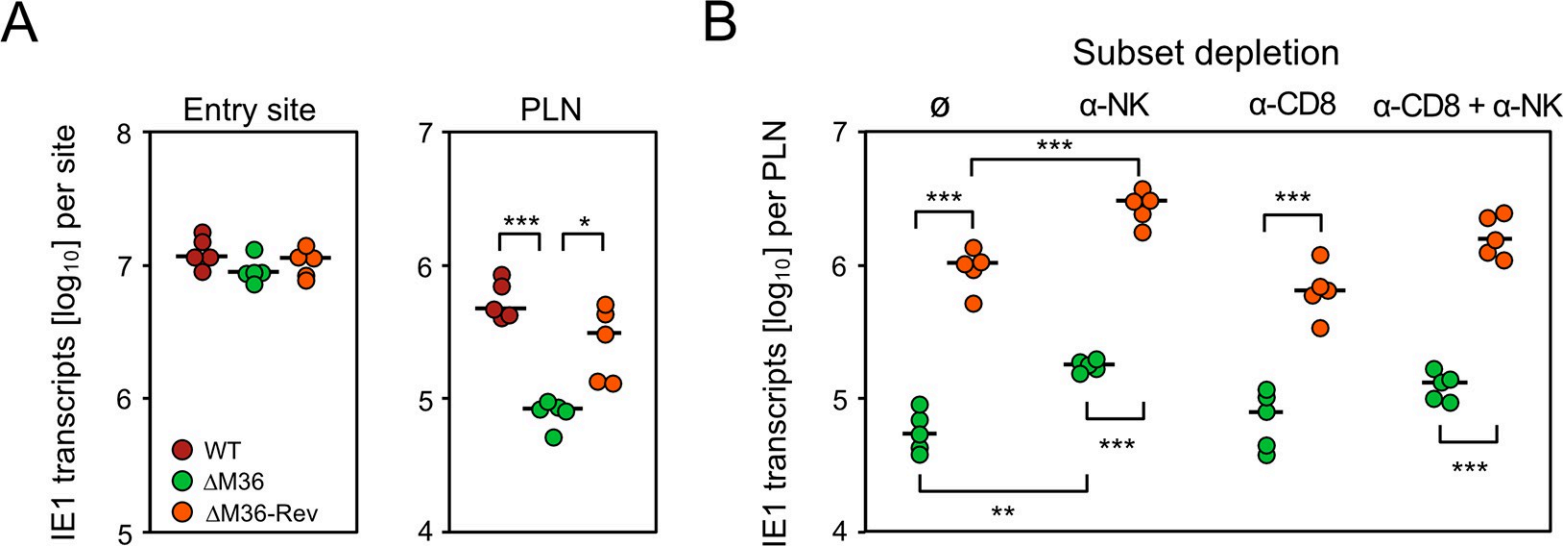
Release from apoptosis blockade by deletion of M36 leads to virus growth attenuation in the PLN but not locally at the viral entry site. (**A**) Level of infection determined by quantitation of IE1 transcripts in plantar (footpad) tissue (left panel) and in the draining RNL, specifically the PLN (right panel), at 48 hours after intra-plantar infection. (**B**) Level of infection determined by quantitation of IE1 transcripts in the PLN at 72 hours after intra-plantar infection. At 24 hours before infection, mice were depleted of NK cells (panel α-NK), of CD8 T cells (panel α-CD8), of both (panel α-CD8 + α-NK), or were left undepleted (panel ∅). Different times of analyses in (**A**) and (**B**) take into account that virus replication precedes the immune response. Infecting viruses are indicated in the internal legend and color-coded as in Fig 2B. Filled circles represent data from 5 mice per experimental group (n = 5) tested individually. Median values are marked. Levels of significance are indicated for group comparisons of interest: P-values (*) <0.05, (**) <0.01, and (***) <0.001.

During evolution, CMVs have acquired inhibitors of programmed cell death to avoid premature death of infected cells before progeny virions are assembled and released for cell-to-cell spread. Accordingly, deletion of these inhibitors should lead to virus growth attenuation. We tested this prediction for the example of M36 (Fig 3). We quantitated spliced immediate-early 1 (IE1) transcripts, which are proportional to the number of infected cells, as a surrogate for the degree of infection. The more direct parameter for viral replication, the copy number of viral DNA, cannot be used in this particular case, because the infection dose of 10^5^ infectious units (plaque-forming units, PFU) equals ~5 x 10^7^ genomic viral DNA molecules (genome-to-PFU ratio of ~500:1) [92,93] that obscure the detection of *de novo* synthesized viral DNA, especially at early times after infection. IE1 transcripts were chosen, because they precede transcription of cell death inhibitor genes in the viral productive cycle.

On day 2 after intra-plantar infection, a time allowing to complete just one round of local viral replication in plantar tissue [94], IE1 gene expression was identical for WT virus and ΔM36 mutant as well as revertant viruses (Fig 3A, left panel). This indicates an equivalent viral capacity to initially infect cells at the entry site and suggests resistance to apoptosis of infected connective tissue cells. In contrast, in the PLN, the M36 deletion mutant was significantly growth-attenuated compared to WT virus and the revertant virus (Fig 3A, right panel), indicating that infected cells in the PLN are susceptible to apoptosis. In accordance with apoptosis of infected cells in the PLN, previous work has shown that mCMV-ΔM36 fails to disseminate from the PLN to downstream organ sites of infection, as shown exemplarily for the liver, unless inhibition of apoptosis is restored by functional M36 trans-complementation upon co-infection with WT virus [76].

Considering the known function of M36 in blocking apoptotic signaling, growth attenuation of the deletion mutant likely reflects reduced virus production due to premature death of infected cells. Nonetheless, we also considered the alternative explanation of an enhanced immune control, selectively of the mutant virus, by viral antigen cross-presentation to CD8 T cells. This idea was refuted, however, by depletion of NK cells, CD8 T cells, or both (Fig 3B). While the depletion of CD8 T cells had no notable effect on early viral replication, the depletion of NK cells indicated some early control by innate immunity, which is in accordance with a previous report on immune control in the PLN after intra-plantar infection of BALB/c mice with mCMV-WT [72]. Of relevance, the difference between mutant and revertant virus was not abolished by any immune cell subset depletion.

In conclusion from this set of data, growth attenuation of mCMV-ΔM36 in the PLN is not caused by an enhanced immune control but is consistent with apoptosis of infected cells preventing virus release and spread.

### Alternative inhibition of apoptosis by blocking FADD signaling reverts the effect of M36 deletion

All data shown so far already strongly supported the conclusion that enhanced priming after infection with mCMV-ΔM36 can be attributed to apoptosis that leads to the release of apoptotic bodies taken up by uninfected pAPCs, in accordance with established knowledge on the mechanism of antigen cross-presentation [95–98]. By *in situ* detection of effector caspase-3 (recall Fig 2A) previous work by Cicin-Sain and colleagues [76] has unequivocally shown that infection with mCMV-ΔM36 leads to apoptosis of infected cells *in vivo*, including liver macrophages, and that this phenotype of effector caspase-3 expression is reversed after infection with mCMV-ΔM36-Rev blocking apoptosis. Although the work by Chaudhry and colleagues [70] did not address the issue of antigen cross-presentation, blockade of apoptosis by hCMV UL36 replacing mCMV M36 in the chimeric virus mCMV^UL36^ is also in line with an involvement of apoptosis.

While all data are best compatible with antigen cross-presentation based on apoptosis being the primary mechanism by which deletion of M36 enhances CD8 T-cell priming, a modulating contribution by a currently unknown additional function of M36 is not formally excluded. Recent work on hCMV UL36 indeed indicated a blockade of IRF3 signaling in addition to its known function of blocking apoptosis [99]. While it is unknown if this function is conserved and applies also to M36, replacement of M36 with UL36 [70] shows at least that the apoptosis phenotype is not affected.

We first sought to test if a virus growth-attenuating mutation in a signaling pathway unrelated to programmed cell death signaling might also enhance CD8 T-cell priming. For this, we chose M27 that binds to STAT2 and is reported to block antiviral type-I/III and type-II interferon signaling [100–102]. In accordance with the literature, deletion of M27 in virus mCMV-ΔM27, which thus cannot evade antiviral type-I/III and type-II interferon functions, led to viral growth attenuation in the PLN (S3A Fig). As a new finding, CD8 T-cell priming was not enhanced but, instead, was even reduced (S3B Fig). This result is essentially what one would have expected, because reduced virus replication and spread should reduce the number of infected cells and thus limit the amount of antigen available for CD8 T-cell priming. This example makes us realize that a virus growth attenuation corresponding to an enhanced instead of a reduced CD8 T-cell response is something special that needs to be explained.

**Fig 4:**
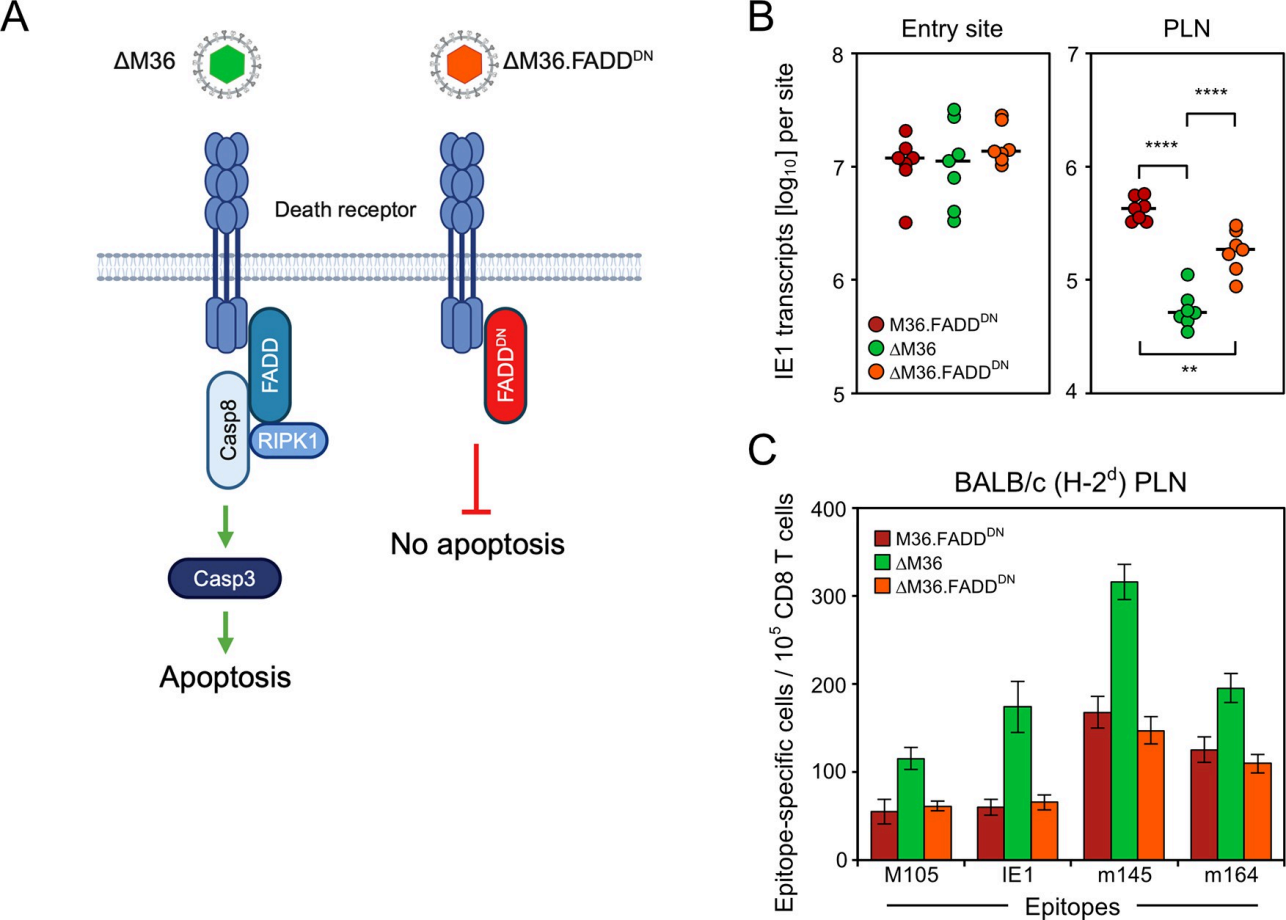
Interruption of apoptotic signaling by virally-encoded dominant-negative FADD functionally reverts virus growth attenuation and the enhancing effect of M36 deletion on the antiviral CD8 T-cell response. (**A**) Sketch illustrating the block in the apoptotic signaling pathway by replacement of cellular FADD with virally-encoded FADD^DN^. (ΔM36) mCMV-ΔM36; virus symbol colored green to indicate the approval of apoptosis by deletion of M36. (ΔM36.FADD^DN^) mCMV-ΔM36.FADD^DN^; virus symbol colored red to indicate the interruption of signaling by FADD^DN^. The sketch was created with BioRender.com. (**B**) Interruption of apoptotic signaling by virally-encoded dominant-negative FADD functionally reverts virus growth attenuation caused by M36 deletion. Levels of infection were determined by quantitation of IE1 transcripts in plantar (footpad) tissue (left panel) and in the draining RLN, the PLN (right panel), at 48 hours (day 2) after intra-plantar infection (day 0) with the viruses indicated in the internal legend. For further information and statistical evaluation, see the legend of Fig 3. (**C**) Interruption of apoptotic signaling by virally-encoded dominant-negative FADD functionally reverts the enhancing effect of M36 deletion on the antiviral CD8 T-cell response. The viral epitope-specific CD8 T-cell response was determined for the PLN on day 7 after intra-plantar infection of BALB/c mice (cohorts of 5 mice) with the viruses indicated in the internal legend. (Green color) apoptosis can take place. (Red colors) apoptosis is blocked. For further information, see the legend of Fig 2B.

A hypothetical contribution of M36 to viral fitness by an unidentified function unrelated to the inhibition of apoptosis has also been an issue in previous work by Cicin-Sain and colleagues [81]. The authors used the elegant approach of converting the apoptosis phenotype of active caspase-3 expression after infection with mCMV-ΔM36 by blocking apoptotic signaling not only by reinsertion of M36 in virus mCMV-ΔM36-Rev, which restitutes both the block in apoptosis and putative other functions of M36, but alternatively also earlier in the signaling cascade by viral expression of a dominant-negative (DN) mutation of FADD (FADD^DN^) restituting the block in apoptosis selectively (Fig 4A). Specifically, FADD^DN^ competes with cellular FADD and reverts the apoptosis phenotype of ΔM36 in cells infected with recombinant virus mCMV-ΔM36.FADD^DN^ [81].

Here we used this approach to reproduce not only the published effect of FADD^DN^ restoring viral fitness in the PLN (Fig 4B), but also expanded on studying its effect on CD8 T-cell priming (Fig 4C). Analogous to the experiment shown in Fig 2B, the CD8 T-cell response was tested in the PLN of BALB/c mice on day 7 after intra-plantar infection. Compared to a control virus expressing M36 and FADD^DN^ (M36.FADD^DN^), the CD8 T-cell response was consistently enhanced by infection with mCMV-ΔM36 for the panel of viral epitopes tested, and this was reversed by expression of FADD^DN^ after infection with virus mCMV-ΔM36.FADD^DN^.

From all this compiled evidence, we conclude that the mechanism by which deletion of M36 increases the CD8 T-cell response is indeed related to enhanced apoptosis.

### Enhancement of apoptosis by deletion of M36 leads to an improved antiviral CD8 T-cell response in terms of response magnitude and specificity repertoire

**Fig 5:**
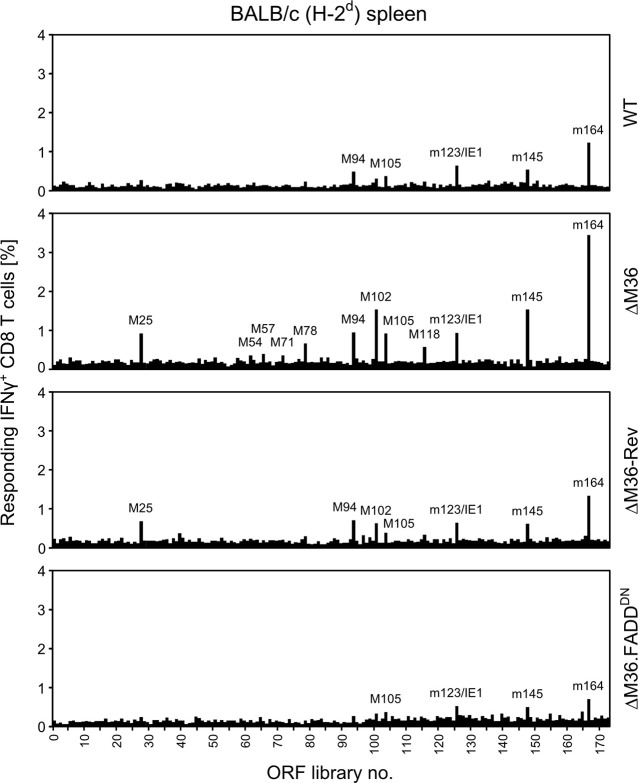
Viral genome-wide screening of the viral antigen-specific CD8 T-cell response in dependence on apoptosis. A viral genome-spanning ORF library of expression plasmids was used to test the overall CD8 T-cell response determined for the spleen on day 7 after intra-plantar infection of BALB/c mice (cohorts of 7 mice) with the viruses indicated. Signals represent frequencies of CD8 T cells activated to express intracellular IFNγ after stimulation with antigenic transfectants. For more prominent signals, the corresponding ORF is named.

So far, we have tested the impact of M36 deletion on the CD8 T-cell response in the PLN of BALB/c mice or of C57BL/6 mice with selected panels of mCMV epitopes for which the corresponding antigenic peptides are known in their amino acid sequence [103,104]. To get an impression of the overall antiviral CD8 T-cell response, including viral epitopes not yet identified in their amino acid sequence, we employed an mCMV genome-wide open reading frame (ORF) library of expression plasmids [103] using responder CD8 T cells derived from the spleen on day 7 after intra-plantar infection of BALB/c mice (recall S2B Fig) with viruses preventing or allowing apoptosis (Fig 5, for the assay setup, see [84,103]). Compared to viruses preventing apoptosis by expression of M36 (WT and ΔM36-Rev) or FADD^DN^ (ΔM36.FADD^DN^), the overall antiviral CD8 T-cell response was higher and broader with the M36 deletion mutant mCMV-ΔM36.

### Enhancement of necroptosis by inactivation of M45 leads to a generally higher and broader antiviral CD8 T-cell response

**Fig 6:**
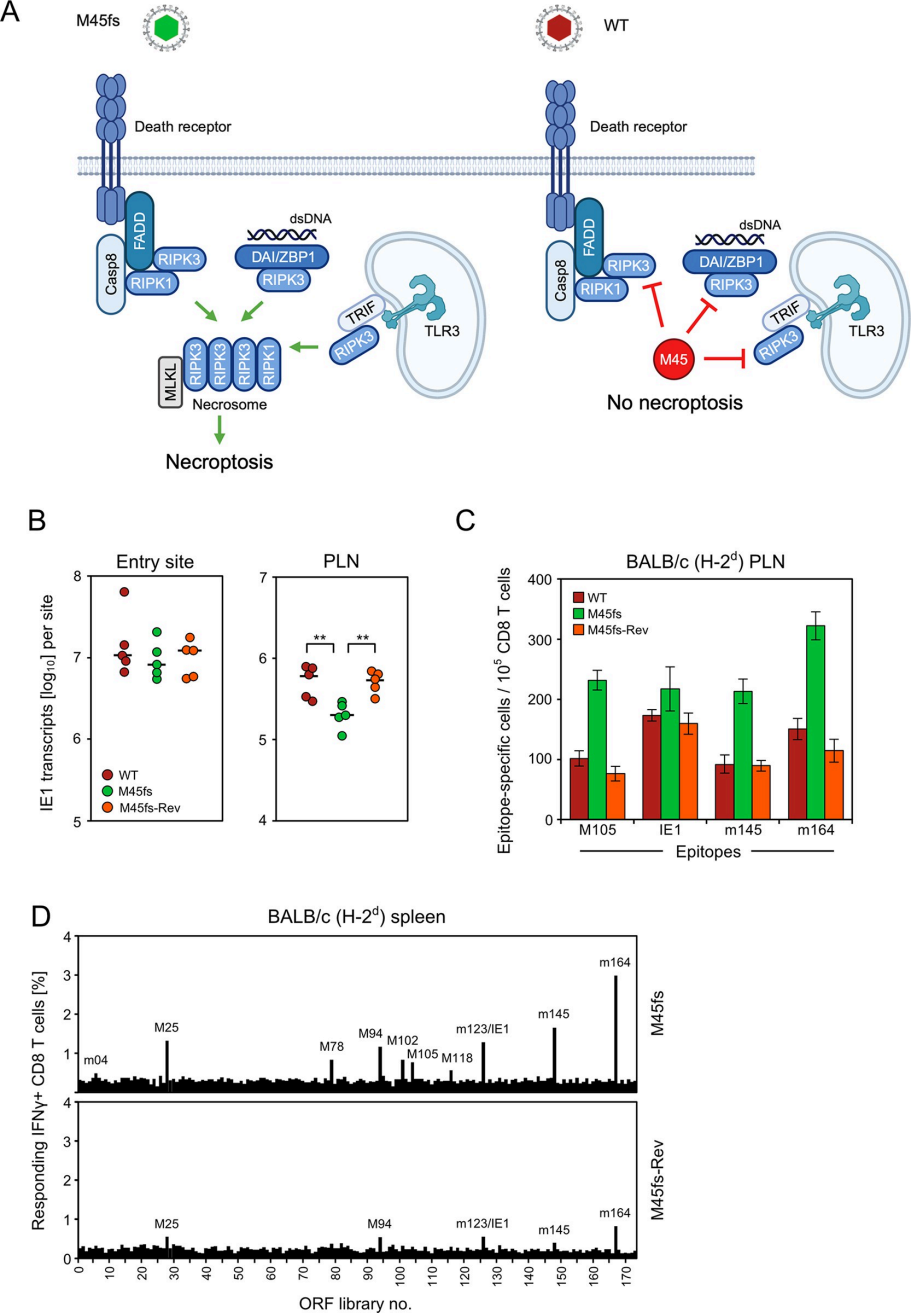
Release from necroptosis blockade by functional deletion of M45 leads to virus growth attenuation and enhances the antiviral CD8 T-cell response. (**A**) Sketch illustrating the block in the necroptotic signaling pathway by mCMV protein M45. (M45fs) mCMV-M45fs; virus symbol colored green to indicate the approval of necroptosis by functional deletion of M45. (WT) mCMV-WT; virus symbol colored red to indicate the inhibition of necroptosis by functional M45. The sketch was created with BioRender.com. (**B**) Release from necroptosis blockade by functional deletion of M45 leads to virus growth attenuation in the PLN but not locally at the viral entry site. Levels of infection were determined by quantitation of IE1 transcripts in plantar (footpad) tissue (left panel) and in the PLN (right panel), at 48 hours (day 2) after intra-plantar infection (day 0) with the viruses indicated in the internal legend. For further information and statistical evaluation, see the legend of Fig 3. (**C**) Release from necroptosis blockade by functional deletion of M45 enhances the antiviral CD8 T-cell response. The viral epitope-specific CD8 T-cell response was determined for the PLN on day 7 after intra-plantar infection of BALB/c mice (cohorts of 5 mice) with the viruses mCMV-WT (WT) expressing functional M45, mCMV-M45fs (M45fs) lacking functional M45, and the revertant virus mCMV-M45fs-Rev (M45fs-Rev) expressing functional M45. (Green color) necroptosis can take place. (Red colors) necroptosis is blocked. For further information, see the legend of Fig 2B. (**D**) ORF library screening of the overall CD8 T-cell response determined for the spleen on day 7 after intra-plantar infection of BALB/c mice (cohorts of 7 mice) with the viruses indicated. For further information see the legend of Fig 5.

We next addressed the question if the impact of programmed cell death on the CD8 T-cell response applies selectively to apoptosis or also to necroptosis (for a scheme, see Fig 6A). The mCMV-encoded inhibitor of RIPK3-signaling M45 prevents the formation of the necroptosis signaling complex, also known as necrosome [57], particularly in macrophages [65]. It is important to note that necroptosis after deletion of M45 comes into charge only when apoptosis is blocked by M36 [17,57,62,67].

We compared viral fitness (Fig 6B) and the CD8 T-cell response to selected viral epitopes (Fig 6C) in the PLN of BALB/c mice on day 7 after intra-plantar infection with the necroptosis-permitting virus mCMV-M45fs, in which M45 is inactivated by a frame-shift mutation [105], and the necroptosis-inhibiting viruses mCMV-WT and mCMV-M45fs-Rev. As it was the case for the apoptosis inhibitor M36 (recall Fig 2A), necroptosis in absence of a functional M45 led to virus growth attenuation in the PLN that was reversed for mCMV-M45fs-Rev expressing M45 (Fig 6B). This analogy between inhibition of apoptosis and inhibition of necroptosis also extends to the CD8 T-cell response that was enhanced compared to the WT virus control for all viral epitopes tested after infection with mCMV-M45fs lacking functional M45, and was reversed to WT levels for mCMV-M45fs-Rev expressing functional M45 (compare Fig 2B with Fig 6C). To get an impression of the overall antiviral CD8 T-cell response, we again used the genome-wide ORF-library of expression plasmids (recall Fig 5) for screening the response by CD8 T cells derived from the spleen of BALB/c mice on day 7 after intra-plantar infection (Fig 6D). The response was found to be enhanced in terms of magnitude and specificity repertoire after infection with mCMV-M45fs compared to mCMV-M45fs-Rev.

### Programmed cell death enhances the CD8 T-cell response by antigen cross-presentation

All data have consistently shown that programmed cell death results in an enhanced antiviral CD8 T-cell response. It is well-established that apoptotic bodies and necroptotic extracellular vesicles can be taken up efficiently by mature, uninfected pAPCs for antigen cross-presentation to prime naïve CD8 T cells [95,106,107]. It was therefore more than reasonable to propose that the enhanced CD8 T-cell response observed after deletion of cell death inhibitors was caused by antigen cross-presentation.

**Fig 7:**
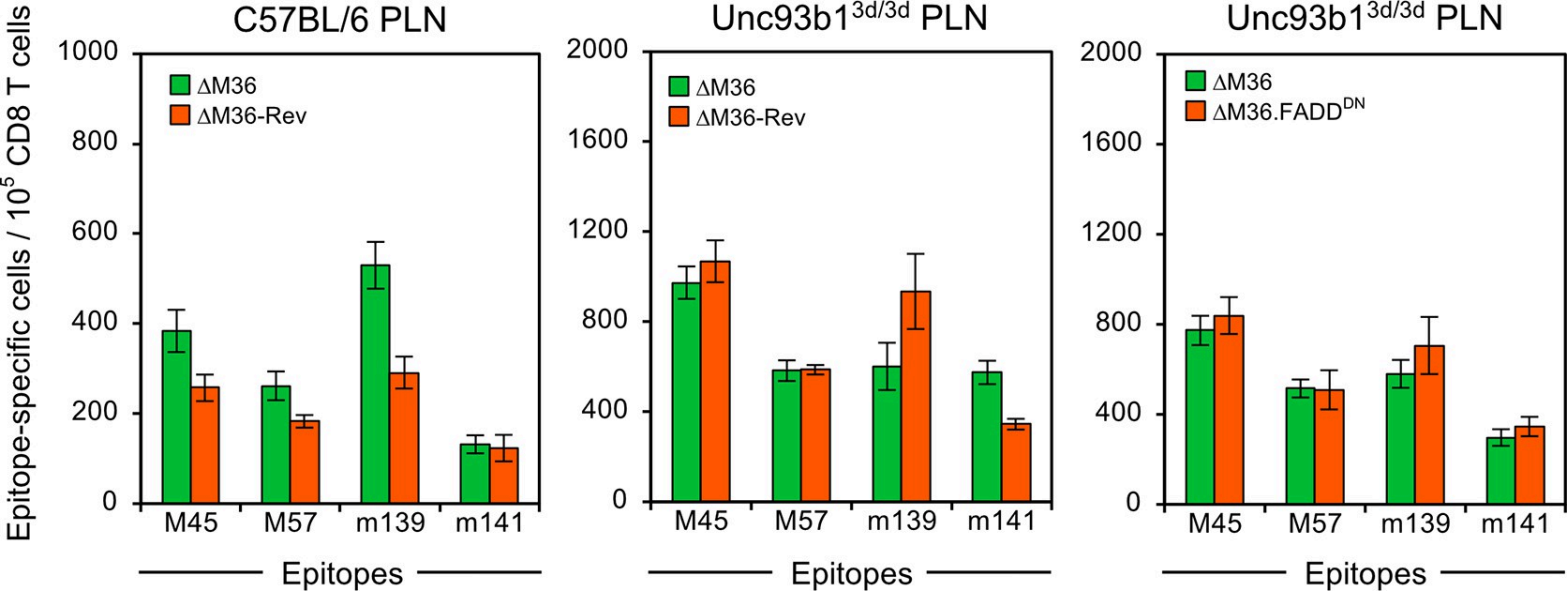
Missing contribution of apoptosis to the CD8 T-cell response in C57BL/6-Unc93b1^3d/3d^ mice genetically unable to cross-present. The viral epitope-specific CD8 T-cell response was determined for the PLN on day 7 after intra-plantar infection of cohorts of 4 mice of WT strain C57BL/6 (left panel) and mutant strain Unc93b1^3d/3d^ (center and right panel) with the viruses indicated. For further information, see the legend of Fig 2B.

If this interpretation applies, the difference in CD8 T-cell priming should not be observable in mouse mutant strain C57BL/6-Unc93b1^3d/3d^ genetically deficient in antigen cross-presentation [52]. Indeed, comparing the CD8 T-cell response in dependence of apoptosis in cross-presentation-competent C57BL/6 mice with that in cross-presentation-deficient Unc93b1^3d/3d^ mice revealed an enhanced priming by mCMV-ΔM36 only in C57BL/6 mice capable to cross-present (Fig 7).

The overall strong CD8 T-cell response to mCMV in Unc93b1^3d/3d^ mice is thus based entirely on direct antigen presentation and shows that it remains the predominant mode of CD8 T-cell priming also when apoptosis can take place. The most important conclusion from these experiments is that programmed cell death does not enhance the CD8 T-cell response when antigen cross-presentation is precluded. Conversely, the results indicate that the enhancement of the response observed in mice that are genetically competent in cross-presentation indeed resulted from cross-presentation as an add-on to an underlying direct antigen presentation.

## Discussion

The way how naïve CD8 T cells become primed to mount a protective antiviral response has long been a matter of debate in CMV immunology. Evidence was presented supporting direct antigen presentation by infected pAPCs as well as antigen cross-presentation by uninfected pAPCs that engulf antigenic material derived from infected cells undergoing programmed cell death and releasing apoptotic bodies or necroptotic extracellular vesicles. However, proponents of one mechanism or the other have used sophisticated experimental protocols or mutant mouse strains to rule out the respective alternative mechanism. Specifically, experimental prevention of direct antigen presentation showed priming by cross-presentation [47,48,108], and prevention of cross-presentation showed priming by direct presentation [51,109]. The bottom line message was that, in principle, both mechanisms can generate antiviral CD8 effector T cells recognizing the same viral epitopes in the same immunodominance hierarchy [47,51]. However, which type of priming prevails in mice competent for both, direct and cross-presentation, has long been an open question.

Using the new strategy to modulate direct antigen presentation by mCMV mutants in which the key immune evasion gene m152 was either deleted or overexpressed, we recently showed that the CD8 T-cell response directly reflects the level of antigen presentation in form of pMHC-I complexes on the surface of infected cells [51,53]. This applied to priming of naïve CD8 T cells in the PLN of immunocompetent mice [51], but also to priming in the medically relevant situation after HCT when CD8 T cells become reconstituted in hematoablated HCT recipients only gradually over time [53]. However, it was not understood why antigen cross-presentation, which can be enforced by particular experimental conditions [47], does not seem to play a significant role under physiological conditions.

Here we addressed this open question by studying mCMV mutants with deletion of the viral cell death inhibitors M36 or M45. For mouse strains genetically capable of antigen cross-presentation, such as BALB/c and C57BL/6, our data show an enhanced CD8 T-cell response to mutant viruses that permit apoptosis or necroptosis. Focusing on M36-governed apoptosis, this response increment is shown to be lost in Unc93b1^3d/3d^ mice genetically deficient in antigen cross-presentation [52] (Fig 7). Thus, viral inhibition of programmed cell death can explain the lack of antigen cross-presentation in normal mice. As a by-product, direct antigen presentation in Unc93b1^3d/3d^ mice was not negatively affected by apoptosis (Fig 7), although premature death of infected pAPCs should reduce direct antigen presentation.

To explain this unexpected finding, we can currently only speculate that priming by direct antigen presentation might be completed before the infected pAPCs get lost. Strikingly, CD8 T-cell priming in Unc93b1^3d/3d^ mice after infection generally results in higher frequencies for all epitopes tested compared to C57BL/6 mice (Figs 2B and 7), a phenomenon that has been observed previously but remained unexplained [51,110]. Unc93b1^3d/3d^ mice show a reduced antiviral cytokine response to mCMV, with low serum levels of IFNγ, IL-12, tumor necrosis factor (TNF), and type-I interferons, resulting in an increased viral load [52,110]. Thus, by increasing the number of infected pAPCs involved in direct antigen presentation, this enhanced viral replication could explain the strong CD8 T-cell response to mCMV in Unc93b1^3d/3d^ mice.

Another topic of discussion is the question of why the "late death" of productively infected cells by cytolysis as the endpoint of the cytopathogenicity of CMVs does not seem to support antigen cross-presentation. A possibility might be that cell debris resulting from cytolytic cell death, unlike apoptotic bodies or necroptotic extracellular vesicles, lacks signals or ligands that facilitate uptake by cross-presenting pAPCs [111,112].

Programmed death of infected cells initiated by the expression of death receptor ligands is an antiviral defense mechanism of the host to prevent viral spread. Thus, from the perspective of viral evolution, acquisition of genes that inhibit apoptosis and necroptosis enables the virus to prevent premature cell death, and thereby allows completion of the viral productive cycle before the infected cells eventually die a “late death” from the cytolytic infection [17].

Previous work on the function of M36 and M45 emphasized their role in "viral fitness" as a pathogenicity factor and determinant of cell tropism [105] by showing “viral attenuation” in case of their deletion [64,65,68,81–83,105,113–116]. Interestingly, in the case of M36 deletion, attenuation is also associated with greater susceptibility to effector CD8 T-cell control, based on a cross-talk between apoptotic signaling and perforin/granzyme-dependent cytotoxicity [66]. In accordance with these studies, we document an early viral growth attenuation in the PLN by deletion of M36 (Figs 3A and 4B) or M45 (Fig 6B), although at least this early effect was not mediated by an antiviral immune control (Fig 3B). As a new aspect, our data add the information that cell death inhibitors contribute to viral spread and pathogenesis not only by preventing premature cell death to allow completion of the viral replication cycle but, in addition, also by preventing antigen cross-presentation leading to a reduced CD8 T-cell response.

We were initially wondering why viral growth attenuation by deletion of M36 or M45 is not seen at the viral entry site, which is the footpad tissue in the case of intra-plantar infection (Figs 3A, 4B and 6B). However, there exists an explanation. Virions administered into the interstitial fluid do not need to spread from infected footpad connective tissue to the RLN after a first round of local infection, but can directly reach the RLN within minutes via afferent lymphatic vessels [73,117]. Therefore, the observed difference in virus growth most likely results from different susceptibility to programmed cell death of the cell types infected in footpad tissue and the PLN. Murine fibroblasts and endothelial cells were reported to undergo apoptosis upon mCMV-ΔM36 infection only after conditioning by tumor necrosis factor α (TNFα) [118]. Our functional data on the lack of an M36 deletion phenotype in the footpad (Figs 3A and 4B) suggest that infected connective tissue fibrocytes or infected endothelial cells in the footpad vasculature are not susceptible to ΔM36-associated apoptosis in an early stage of infection. In this context, it is important to recall that M36 and M45 were originally both found to operate in infected macrophages [64,82,83], and M36 was found to block apoptosis also in DCs [118].

After intravenous infection of immunocompromised BALB/c mice, mCMV-ΔM36 was found to replicate in liver tissue, and apoptosis was detected *in situ* by staining of active caspase-3 in Kupffer cells, which are the liver macrophages, but most prominently also in hepatocytes, which are the liver parenchymal cells [81].

Importantly, macrophages and DCs are natural sources of TNFα that trigger apoptosis in an autocrine fashion after infection with mCMV-ΔM36 [118]. In this context, it is important to note that virions reaching an RLN via the lymphatics localize to the subcapsular sinus (SCS) where they infect SCS-lining macrophages and DCs ([117] and references therein). Moreover, infected macrophages are known determinants of viral pathogenesis [119], and in a related model, mCMV was found to infect CD169^+^ SCS macrophages in mediastinal lymph nodes after systemic infection [120]. From all this evidence we propose that growth attenuation of virus mCMV-ΔM36 in the PLN is caused by apoptosis of infected SCS macrophages and, likely, also SCS DCs. Importantly, the same rules apply to necroptosis by deletion of M45 under the condition that M36 is expressed to prevent apoptosis (Fig 6B).

Our key result of CD8 T-cell priming by antigen cross-presentation, dependent upon either apoptosis or necroptosis after deletion of programmed cell death inhibitors M36 or M45, respectively, is based on consistent data throughout. Uninfected pAPCs that take up apoptotic bodies or necroptotic extracellular vesicles, respectively, in the PLN following intra-plantar infection can be cross-presentation-competent SCS DCs, for instance, CD8^+^CD11c^+^ DCs [54,55], but also SCS macrophages identified more recently as being also capable to cross-present [121].

One should always keep in mind that studying viral mutants does not aim to understand the biology of the mutants but to learn about the evolutionary successful WT virus. While our data have shown that mCMV-specific CD8 T cells can be primed by antigen cross-presentation, provided that apoptosis or necroptosis are facilitated by deletion of the respective viral inhibitor, the relevant message for understanding the CD8 T-cell response to WT virus is the reverse. CD8 T-cell priming to mCMV-WT occurs predominantly, if not exclusively, by direct antigen presentation, because viral cell death inhibitors prevent a significant contribution of antigen cross-presentation. While antigen cross-presentation would have been in the host’s interest, ensuring viral replicative capacity by simultaneously blocking premature cell death and antigen cross-presentation was likely a driver of selection in CMV evolution.

The mode of CMV antigen presentation for CD8 T-cell priming was a long-debated fundamental question in CMV immunology. While our recent work has identified direct antigen presentation as the canonical mechanism [51,53], the reason why antigen cross-presentation plays a minor role was not known. This knowledge gap has now been closed by demonstrating the prevention of antigen cross-presentation by viral inhibitors of programmed cell death.

## Materials and methods

### Ethics statement

Animal experiments were performed in accordance with the national animal protection law (Tierschutzgesetz (TierSchG)), animal experiment regulations (Tierschutz-Versuchstierverordnung (TierSchVersV)), and the recommendations of the Federation of European Laboratory Animal Science Association (FELASA). The experiments were approved by the ethics committee of the Landesuntersuchungsamt Rheinland-Pfalz, permission numbers 17-07-04/051-62 and 177-07/G09-1-004.

### Cells, viruses, and mice

P815 (No. TIB-64, haplotype H-2^d^) and EL4 (No. TIB-39, haplotype H-2^b^) cells were obtained from the American Type Culture Collection (ATCC) and cultivated in RPMI supplemented with 5% fetal calf serum (FCS) and antibiotics, or in DMEM with 10% FCS and antibiotics, respectively. Primary murine embryo fibroblasts (MEF) were cultivated in MEM supplemented with 10% FCS and antibiotics.

Virus derived from BAC plasmid pSM3fr [122] was used as “wild-type (WT)” virus, mCMV-WT. Recombinant viruses mCMV-ΔM36 [64] and mCMV-ΔM36-Rev [81], as well as mCMV-M45.BamX and mCMV-M45.BamX-Rev [105] have been described previously. Virus mCMV-M45.BamX carries a frameshift mutation in M45 [105] and is, for simplicity, renamed mCMV-M45fs in this study. Accordingly, the corresponding revertant virus is renamed mCMV-M45fs-Rev. Recombinant viruses mCMV-ΔM36-FADD^DN^ and mCMV-WT-FADD^DN^ [81], here renamed mCMV-ΔM36.FADD^DN^ and mCMV-M36.FADD^DN^, were used as functional revertants of mCMV-ΔM36. The recombinant virus mCMV-ΔM27 [100] carries a deletion of the open reading frame M27 encoding a STAT2-antagonist [100] and was used as control virus that is attenuated by a mechanism unrelated to programmed cell death [123].

BALB/c and C57BL/6 mice were bred and housed under specified-pathogen-free (SPF) conditions in the Translational Animal Research Center (TARC) at the University Medical Center of the Johannes Gutenberg-University Mainz, Germany. C57BL/6-Unc93b1^3d/3d^ (briefly Unc93b1^3d/3d^) mice [52] were bred and housed at the central animal facility of the HZI Braunschweig, Germany.

Throughout the work, infection of mice was performed by injection of 1 x 10^5^ PFU of the indicated viruses into the left hind footpad.

### Depletion of leukocyte subsets *in vivo*

Depletion of NK cells or CD8 T cells was performed 24 h prior to infection by i.v. injection of 25μl rabbit antiserum directed against asialo-GM1 (catalog no. 986–100001; Wako Chemicals, Osaka, Japan) or of 1mg purified antibody directed against CD8 (clone YTS169.4), respectively [72]. Macrophage depletion was performed by intra-plantar injection of 50μl clodronate liposomes (catalog no. C-005; ClodronateLiposomes.com, Haarlem, The Netherlands) or control liposomes (catalog no. P-005; ClodronateLiposomes.com) 3 days prior to infection.

### Quantification of viral transcription in infected tissue

Spliced IE1 (m123/ie1) transcripts were quantitated from total RNA extracted from infected lymph nodes [72] or infected footpads using the RNeasy Lipid Tissue Mini Kit (catalog no. 74804 QIAGEN). 500 ng RNA was used as template for RT-qPCR performed with splice product-specific primers and probes. Absolute quantification of IE1 using *in vitro* transcripts as standard has been described previously [94]. Note that transcription is the more sensitive measure of infection compared to the classical PFU assay and, unlike quantitation of viral genomes, it is not obscured by input virion DNA.

### Peptides

Custom peptide synthesis to a purity of > 80% was performed by JPT Peptide Technologies (Berlin, Germany). Synthetic peptides representing antigenic peptides in mouse haplotype H-2^b^ were M38 (SSPPMFRVP), M45 (HGIRNASFI), M57 (SCLEFWQRV), m139 (TVYGFCLL), and m141 (VIDAFSRL**)** [103,124]. Those for mouse haplotype H-2^d^ were M105 (TYWPVVSDI), m123/IE1 (YPHFMPTNL), m145 (CYYASRTKL), and m164 (AGPPRYSRI) [104]. The synthetic peptides were used for exogenous loading of stimulator cells in the ELISpot assay.

### ELISpot assay

An interferon gamma (IFNγ) enzyme-linked immunospot (ELISpot) assay was performed for quantification of IFNγ-secreting CD8 T cells after sensitization by peptide-loaded stimulator cells. Frequencies of mCMV-specific CD8 T cells were determined by incubation of graded numbers of immunomagnetically-purified CD8 T cells, derived from the PLN or the spleen, with stimulator cells (P815/H-2^d^ or EL4/H-2^b^, as it applied) that were exogenously loaded with synthetic peptides at a saturating concentration of 10^-7^M [72]. Spots were counted automatically based on standardized criteria using Immunospot S4 Pro Analyzer (CTL, Shaker Heights, OH, USA) and CTL-Immunospot software V5.1.36. Frequencies (most probable numbers) of IFNγ-secreting cells and the corresponding 95% confidence intervals were calculated by intercept-free linear regression analysis using Mathematica, version 8.0.4 [72,125].

### Genome-wide ORF library screening by intracellular cytokine assay

An mCMV ORF library of expression plasmids spanning the entire mCMV genome [103] was used for ORF-specific stimulation of *ex vivo* isolated CD8 T cells with transfected SV-40 fibroblasts, followed by cytofluorometric (CFM) detection of intracellular IFNγ. The principle and the method of the ORF library screening have been published previously [103,126].

### Statistical analysis

To evaluate statistical significance of differences between two independent sets of data, the unpaired t-test with Welch’s correction of unequal variances was used. Differences were considered statistically significant for P-values (*) <0.05, (**) <0.01, and (***) <0.001. Calculations were performed with Graph Pad Prism 10.1 (Graph Pad Software, San Diego, CA). In cohort analyses of viral epitope-specific CD8 T cells by the ELISpot assay, differences are considered statistically significant if 95% confidence intervals do not overlap.

## Supporting information

S1 Fig Contribution of macrophages to the virus-specific CD8 T-cell response. Viral epitope-specific CD8 T-cell response determined for the spleen on day 7 after intra-plantar infection of BALB/c mice (cohorts of 5 mice) with mCMV-WT. (Grey shaded bars) depletion of macrophages by clodronate liposomes, (open bars) control group left undepleted. For further information, see the legend of Fig 2B.
(TIFF)

S2 Fig Time course of the viral epitope-specific CD8 T-cell response dependent on presence or absence of apoptosis. (**A**) Acute viral epitope-specific CD8 T-cell response was monitored daily for the PLN between day 2 and day 7 after intra-plantar infection of BALB/c mice. (**B**) Acute and memory viral epitope-specific CD8 T-cell responses were determined for the spleen at the indicated times after intra-plantar infection of BALB/c mice. Mice were infected with mCMV-ΔM36 (ΔM36) lacking M36 or with the revertant virus mCMV-ΔM36-Rev expressing M36. (Green color) apoptosis can take place. (Red color) apoptosis is blocked. For further information, see the legend of Fig 2B.
(TIFF)

S3 Fig Permission of STAT2 signaling by deletion of M27 leads to viral growth attenuation corresponding to a reduction of the antiviral CD8 T-cell response. (**A**) Deletion of M27 leads to virus growth attenuation in the PLN but not at the viral entry site. Levels of infection were determined by quantitation of IE1 transcripts in plantar (footpad) tissue (left panel) and in the draining RLN, the PLN (right panel), at 48 hours (day 2) after intra-plantar infection (day 0). For further information and statistical evaluation, see the legend of Fig 3. (**B**) Permission of STAT2 signaling by deletion of M27 leads to a reduction in the antiviral CD8 T-cell response. The viral epitope-specific CD8 T-cell response was determined for the PLN on day 7 after intra-plantar infection of BALB/c mice (cohorts of 5 mice). For further information, see the legend of Fig 2B. Mice were infected either with virus mCMV-WT (WT) expressing M27 that antagonizes STAT2, or with virus mCMV-ΔM27 (ΔM27) that lacks M27 and thus cannot interfere with STAT2 signaling.
(TIFF)

S1 Data Data that underlies this paper. (PDF)
