# Supplementary figures and images for: Cytomegalovirus inhibitors of programmed cell death restrict antigen cross-presentation in the priming of antiviral CD8 T cells
Source: PLoS Pathog. 2024 Aug 15;20(8):e1012173. doi: 10.1371/journal.ppat.1012173 (PMC11349235; doi:10.1371/journal.ppat.1012173)

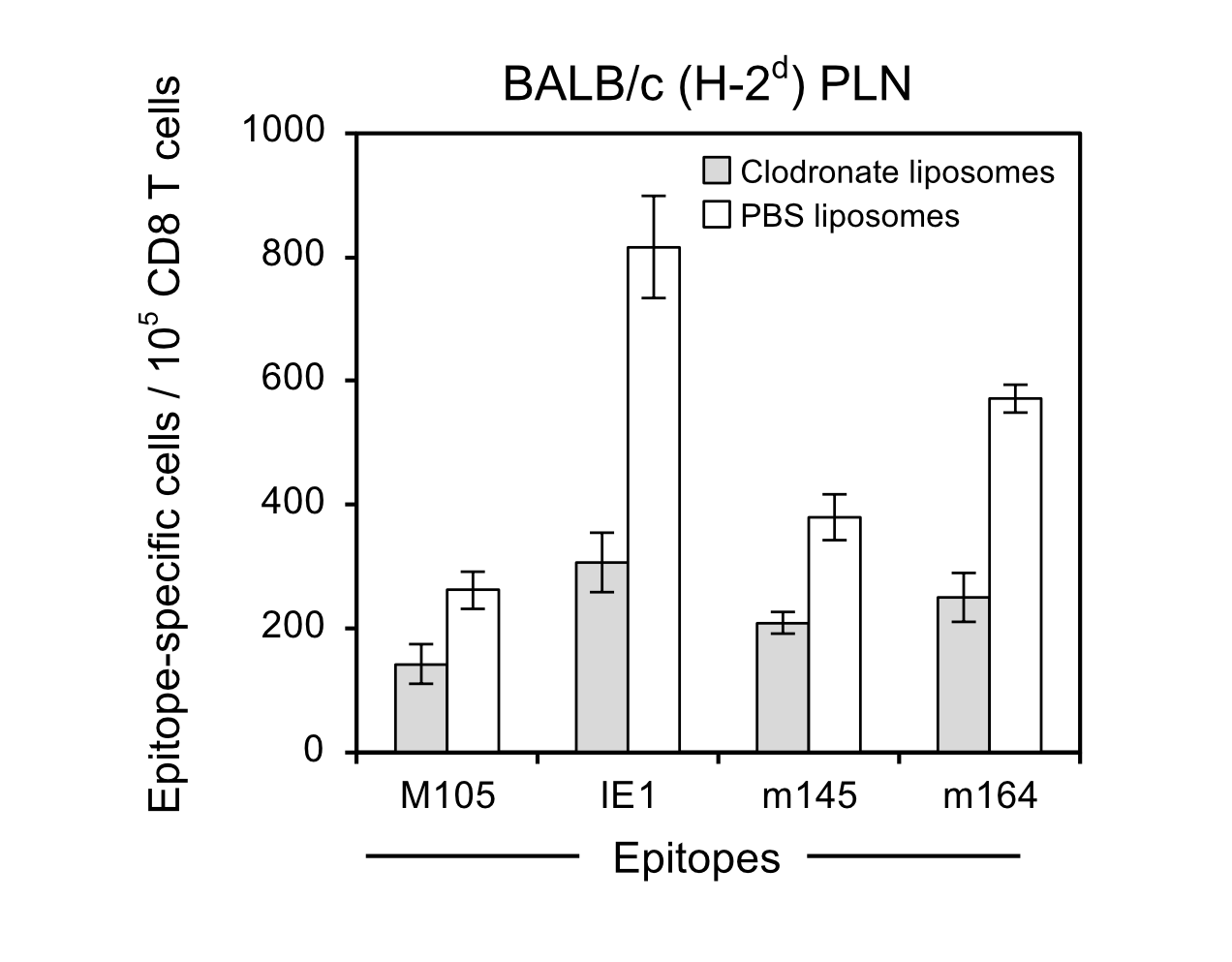

Supplement: S1 Fig — Viral epitope-specific CD8 T-cell response determined for the spleen on day 7 after intra-plantar infection of BALB/c mice (cohorts of 5 mice) with mCMV-WT. (Grey shaded bars) depletion of macrophages by clodronate liposomes, (open bars) control group left undepleted. For further information, see the legend of Fig 2B. (TIFF) [file ppat.1012173.s001.tiff]

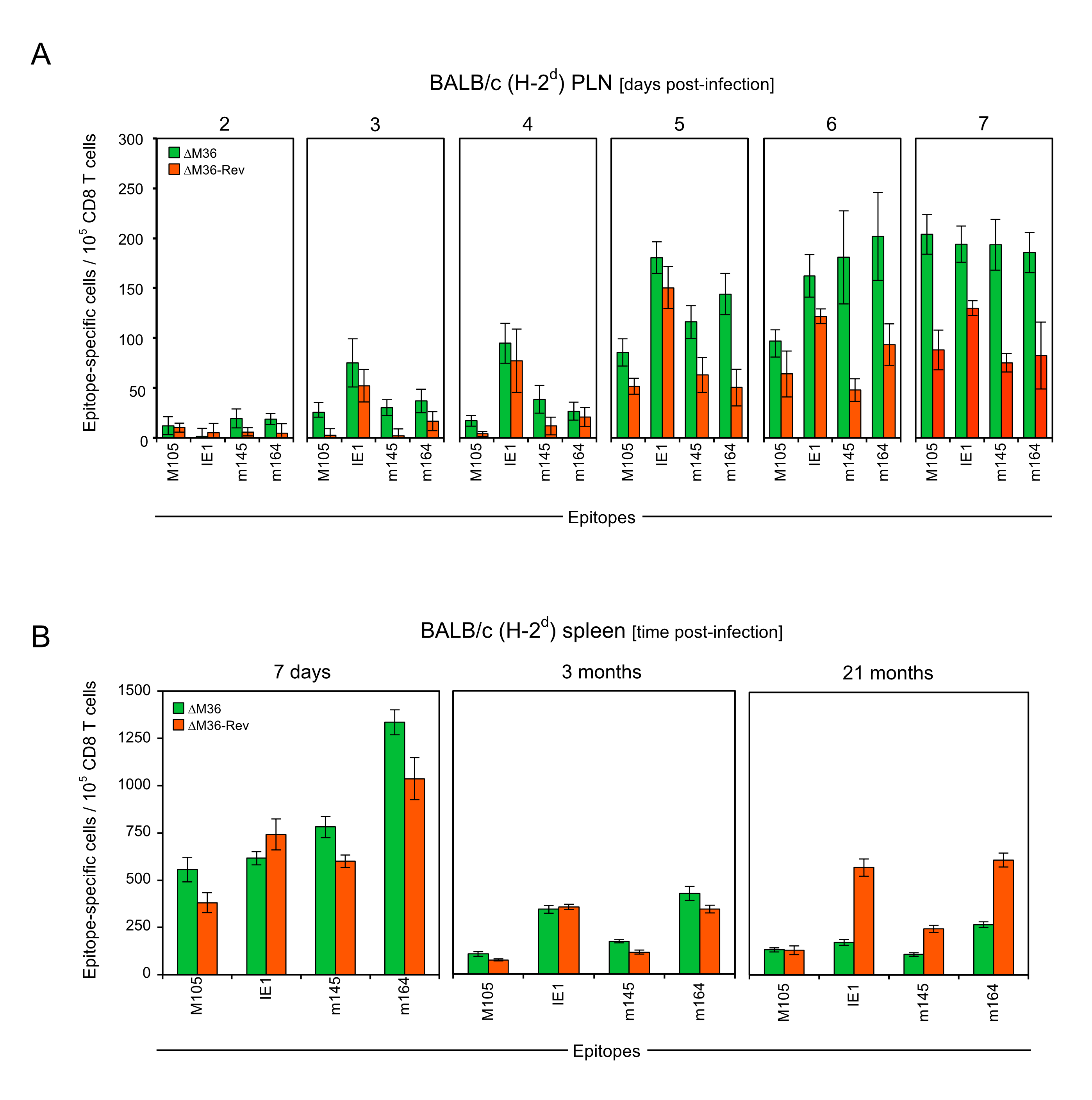

Supplement: S2 Fig — (A) Acute viral epitope-specific CD8 T-cell response was monitored daily for the PLN between day 2 and day 7 after intra-plantar infection of BALB/c mice. (B) Acute and memory viral epitope-specific CD8 T-cell responses were determined for the spleen at the indicated times after intra-plantar infection of BALB/c mice. Mice were infected with mCMV-ΔM36 (ΔM36) lacking M36 or with the revertant virus mCMV-ΔM36-Rev expressing M36. (Green color) apoptosis can take place. (Red color) apoptosis is blocked. For further information, see the legend of Fig 2B. (TIFF) [file ppat.1012173.s002.tiff]

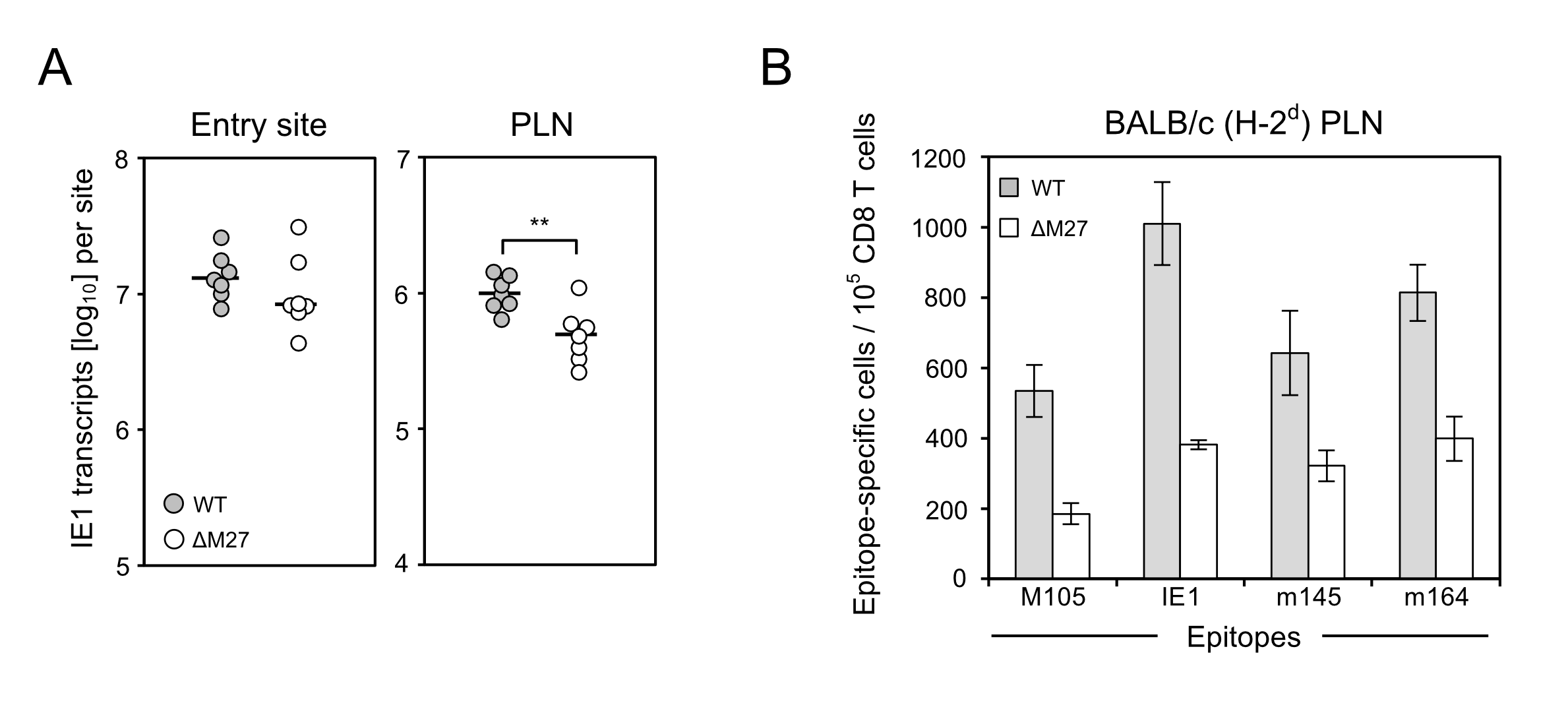

Supplement: S3 Fig — (A) Deletion of M27 leads to virus growth attenuation in the PLN but not at the viral entry site. Levels of infection were determined by quantitation of IE1 transcripts in plantar (footpad) tissue (left panel) and in the draining RLN, the PLN (right panel), at 48 hours (day 2) after intra-plantar infection (day 0). For further information and statistical evaluation, see the legend of Fig 3. (B) Permission of STAT2 signaling by deletion of M27 leads to a reduction in the antiviral CD8 T-cell response. The viral epitope-specific CD8 T-cell response was determined for the PLN on day 7 after intra-plantar infection of BALB/c mice (cohorts of 5 mice). For further information, see the legend of Fig 2B. Mice were infected either with virus mCMV-WT (WT) expressing M27 that antagonizes STAT2, or with virus mCMV-ΔM27 (ΔM27) that lacks M27 and thus cannot interfere with STAT2 signaling. (TIFF) [file ppat.1012173.s003.tiff]
