# Supplementary material for: Cytomegalovirus inhibitors of programmed cell death restrict antigen cross-presentation in the priming of antiviral CD8 T cells
Source: PLoS Pathog. 2024 Aug 15;20(8):e1012173. doi: 10.1371/journal.ppat.1012173 (PMC11349235; doi:10.1371/journal.ppat.1012173)
Supplement: S1 Data — (PDF) [file ppat.1012173.s004.pdf]

Fig 2

WT

Cell No	IE1	m164	m145	M105
10000	19	20	21	8
10000	24	16	13	9
10000	14	16	19	8
25000	46	36	32	27
25000	37	35	36	13
25000	45	44	32	19
50000	79	78	76	48
50000	75	99	68	45
50000	86	86	62	33

ΔM36

Cell No	IE1	m164	m145	M105
10000	22	27	32	12
10000	33	32	34	12
10000	31	34	38	18
25000	76	86	90	34
25000	62	70	77	26
25000	55	78	80	30
50000	142	119	119	59
50000	126	124	122	81
50000	146	127	125	59

ΔM36rev

Cell No	IE1	m164	m145	M105
10000	11	27	15	9
10000	13	20	19	6
10000	8	13	15	7
25000	25	68	44	31
25000	32	48	29	22
25000	27	49	33	22
50000	46	97	70	43
50000	65	89	50	43
50000	54	74	61	40

WT

Cell No	M45	m139	M57	M38
10000	26	17	17	14
10000	25	21	30	13
10000	27	16	20	9
25000	57	57	38	26
25000	51	49	31	25
25000	77	70	61	35
50000	97	93	78	39
50000	115	101	71	54
50000	119	98	76	42

ΔM36

Cell No	M45	m139	M57	M38
10000	48	44	43	13
10000	39	43	37	13
10000	51	44	61	22
25000	111	87	72	51
25000	98	101	71	54
25000	117	121	80	58
50000	136	194	115	123
50000	167	177	109	81
50000	158	201	118	80

ΔM36rev

Cell No	M45	m139	M57	M38
10000	27	18	17	13
10000	23	39	17	16
10000	31	23	26	15
25000	59	80	45	32
25000	57	69	55	30
25000	105	53	51	31
50000	118	124	70	65
50000	107	131	92	57
50000	115	114	81	56

Fig 3

A

Entry site			Lymph node		
**WT**	**Δm36**	**Δm36rev**	**WT**	**Δm36**	**Δm36rev**
1,16E+07	1,32E+07	1,14E+07	4,03E+05	8,64E+04	3,08E+05
1,77E+07	8,87E+06	8,39E+06	8,56E+05	8,17E+04	1,36E+05
1,16E+07	8,73E+06	1,16E+07	7,06E+05	8,38E+04	1,30E+05
1,51E+07	8,81E+06	7,72E+06	4,68E+05	5,19E+04	4,35E+05
8,99E+06	7,27E+06	1,42E+07	4,26E+05	9,55E+04	5,08E+05

B

ø		α-NK		α-CD8		α-CD8 + α-NK	
**Δm36**	**Δm36rev**	**Δm36**	**Δm36rev**	**Δm36**	**Δm36rev**	**Δm36**	**Δm36rev**
7,01E+04	9,32E+05	1,89E+05	3,73E+06	7,88E+04	6,40E+05	1,67E+05	2,47E+06
4,34E+04	1,36E+06	1,68E+05	2,44E+06	3,74E+04	3,38E+05	1,00E+05	2,26E+06
5,45E+04	1,02E+06	1,79E+05	3,04E+06	1,02E+05	1,18E+06	1,33E+05	1,26E+06
9,19E+04	1,06E+06	1,55E+05	1,78E+06	4,45E+04	5,90E+05	1,41E+05	1,56E+06
3,82E+04	5,23E+05	1,98E+05	3,05E+06	1,15E+05	6,90E+05	9,40E+04	1,09E+06

Fig 4

B

entry side			lymph node		
**WT-FADD^{DN}**	**Δm36**	**Δm36-FADD^{DN}**	**WT-FADD^{DN}**	**Δm36**	**Δm36-FADD^{DN}**
2,09E+07	3,34E+06	2,86E+07	5,61E+05	4,81E+04	2,72E+05
3,20E+06	1,13E+07	1,42E+07	4,45E+05	1,11E+05	2,02E+05
1,19E+07	4,02E+06	1,32E+07	4,26E+05	4,36E+04	8,87E+04
1,46E+07	1,28E+07	1,03E+07	3,29E+05	3,51E+04	1,87E+05
1,07E+07	2,77E+07	1,16E+07	3,29E+05	5,15E+04	3,02E+05
9,41E+06	3,22E+07	2,61E+07	3,56E+05	6,64E+04	1,25E+05
1,19E+07	8,02E+06	1,36E+07	5,72E+05	5,42E+04	1,68E+05

C

WT-FADD^{DN}					Δm36					Δm36-FADD^{DN}				
**Cell No**	**IE1**	**m164**	**m145**	**M105**	**Cell No**	**IE1**	**m164**	**m145**	**M105**	**Cell No**	**IE1**	**m164**	**m145**	**M105**
3560	3	5	9	1	4910	4	3	5	1	5680	20	8	17	3
3560	8	2	2	2	4910	5	6	13	0	5680	15	14	17	2
3560	1	6	8	1	4910	6	4	8	3	5680	11	17	14	5
8890	7	10	12	3	12300	12	18	18	7	14200	28	25	33	14
8890	8	12	12	3	12300	8	11	14	6	14200	23	32	45	17
8890	6	7	11	5	12300	6	15	9	6	14200	23	26	29	11
17800	32	19	32	13	24600	17	27	39	15	28400	37	62	95	38
17800	22	21	33	8	24600	14	29	32	17	28400	44	51	90	30
17800	32	26	25	6	24600	16	24	38	13	28400	60	51	89	30

Fig 5

Library no	Gated in %<br>WT	ΔM36	ΔM36-Rev	ΔM36.FADD^{DN}
2	0,13	0,12	0,24	0,15
1	0,11	0,20	0,12	0,08
3	0,16	0,26	0,22	0,10
4	0,23	0,15	0,25	0,07
5	0,19	0,14	0,17	0,06
6	0,15	0,31	0,29	0,14
7	0,14	0,20	0,19	0,16
8	0,08	0,23	0,2	0,12
9	0,10	0,23	0,18	0,11
10	0,10	0,13	0,13	0,13
11	0,13	0,14	0,2	0,14
12	0,22	0,15	0,19	0,13
13	0,14	0,22	0,12	0,12
14	0,10	0,15	0,12	0,14
15	0,07	0,16	0,19	0,14
16	0,18	0,23	0,24	0,14
17	0,10	0,21	0,18	0,21
18	0,11	0,22	0,25	0,11
19	0,16	0,16	0,12	0,13
20	0,11	0,25	0,17	0,15
21	0,09	0,26	0,18	0,14
22	0,12	0,19	0,26	0,21
23	0,12	0,16	0,14	0,11
24	0,20	0,11	0,16	0,16
25	0,15	0,24	0,11	0,09
26	0,11	0,18	0,16	0,18
27	0,16	0,18	0,12	0,14
28	0,28	0,92	0,69	0,24
29	0,11	0,17	0,19	0,14
30	0,14	0,15	0,19	0,11
31	0,09	0,21	0,18	0,07
32	0,12	0,25	0,18	0,17
33	0,11	0,27	0,17	0,17
34	0,10	0,23	0,18	0,12
35	0,06	0,15	0,21	0,16
36	0,17	0,19	0,22	0,23
37	0,18	0,24	0,12	0,15
38	0,11	0,16	0,25	0,12
39	0,21	0,19	0,17	0,19
40	0,20	0,21	0,38	0,15
41	0,18	0,18	0,26	0,15
42	0,12	0,15	0,16	0,07
43	0,16	0,16	0,21	0,09
44	0,08	0,18	0,16	0,10
45	0,04	0,17	0,16	0,26
46	0,12	0,28	0,25	0,22
47	0,10	0,14	0,16	0,16
48	0,14	0,24	0,14	0,11

Fig 5

49	0,19	0,19	0,14	0,16
50	0,11	0,21	0,18	0,12
51	0,15	0,31	0,18	0,18
52	0,18	0,17	0,17	0,12
53	0,13	0,19	0,26	0,17
54	0,15	0,15	0,18	0,12
55	0,20	0,08	0,13	0,15
56	0,14	0,12	0,19	0,16
57	0,13	0,15	0,15	0,16
58	0,15	0,29	0,12	0,10
59	0,08	0,23	0,17	0,20
60	0,10	0,20	0,22	0,15
61	0,16	0,14	0,19	0,11
62	0,14	0,36	0,24	0,17
63	0,21	0,24	0,2	0,10
181	0,12	0,15	0,21	0,13
64	0,13	0,18	0,14	0,24
66	0,14	0,39	0,2	0,14
67	0,14	0,18	0,15	0,08
68	0,12	0,21	0,2	0,19
69	0,10	0,10	0,2	0,12
70	0,12	0,19	0,18	0,15
71	0,10	0,20	0,2	0,13
72	0,13	0,36	0,21	0,11
73	0,16	0,17	0,15	0,09
74	0,14	0,15	0,18	0,19
75	0,10	0,16	0,11	0,09
76	0,09	0,19	0,15	0,12
77	0,10	0,27	0,13	0,10
78	0,12	0,21	0,23	0,07
79	0,23	0,66	0,3	0,15
80	0,09	0,18	0,11	0,09
81	0,10	0,19	0,1	0,19
82	0,08	0,18	0,11	0,15
83	0,11	0,18	0,17	0,17
84	0,09	0,31	0,1	0,20
85	0,13	0,20	0,09	0,11
86	0,07	0,31	0,13	0,12
87	0,11	0,16	0,17	0,10
88	0,16	0,17	0,25	0,15
89	0,09	0,20	0,21	0,12
90	0,15	0,21	0,12	0,08
91	0,16	0,14	0,14	0,12
92	0,16	0,17	0,13	0,10
93	0,18	0,12	0,23	0,10
94	0,49	0,95	0,71	0,27
95	0,14	0,28	0,19	0,12
96	0,11	0,18	0,1	0,08
97	0,14	0,23	0,33	0,12
98	0,13	0,16	0,13	0,18
99	0,15	0,13	0,28	0,20

Fig 5

101	0,21	0,27	0,19	0,18
102	0,31	1,53	0,63	0,33
103	0,11	0,20	0,14	0,14
104	0,10	0,20	0,13	0,21
105	0,37	0,92	0,39	0,37
106	0,12	0,15	0,13	0,14
107	0,08	0,17	0,15	0,27
108	0,13	0,20	0,18	0,19
110	0,15	0,22	0,19	0,21
111	0,15	0,21	0,16	0,16
112	0,18	0,20	0,16	0,15
113	0,10	0,18	0,22	0,27
114	0,22	0,21	0,19	0,17
115	0,18	0,15	0,15	0,23
116	0,14	0,18	0,16	0,16
117	0,13	0,18	0,23	0,26
118	0,23	0,57	0,34	0,22
119	0,11	0,15	0,19	0,18
120	0,10	0,17	0,13	0,14
121	0,19	0,16	0,22	0,15
122	0,07	0,19	0,22	0,27
123	0,16	0,19	0,12	0,23
124	0,09	0,22	0,23	0,21
125	0,13	0,19	0,16	0,25
126	0,17	0,21	0,25	0,25
127	0,12	0,22	0,12	0,16
129	0,65	0,94	0,65	0,53
133	0,16	0,15	0,2	0,29
134	0,09	0,24	0,24	0,28
135	0,11	0,18	0,21	0,23
136	0,11	0,23	0,2	0,30
137	0,16	0,17	0,18	0,21
138	0,14	0,18	0,19	0,22
139	0,17	0,25	0,25	0,16
140	0,16	0,20	0,23	0,27
141	0,20	0,25	0,17	0,22
142	0,26	0,26	0,21	0,19
143	0,11	0,23	0,23	0,31
144	0,16	0,14	0,16	0,14
145	0,15	0,20	0,14	0,14
146	0,19	0,14	0,12	0,34
147	0,24	0,20	0,17	0,23
148	0,18	0,07	0,11	0,26
149	0,22	0,26	0,24	0,13
150	0,14	0,15	0,19	0,14
151	0,11	0,26	0,22	0,25
152	0,22	0,14	0,15	0,22
153	0,21	0,07	0,16	0,18
154	0,54	1,53	0,62	0,50
155	0,12	0,24	0,24	0,25
156	0,18	0,08	0,17	0,13

Fig 5

157	0,26	0,22	0,13	0,23
158	0,09	0,22	0,18	0,19
159	0,16	0,16	0,16	0,16
160	0,11	0,28	0,27	0,19
161	0,16	0,16	0,16	0,33
162	0,08	0,18	0,24	0,16
163	0,12	0,23	0,26	0,17
164	0,14	0,33	0,22	0,30
165	0,16	0,17	0,17	0,21
166	0,18	0,19	0,18	0,18
167	0,15	0,17	0,18	0,17
168	0,14	0,18	0,21	0,20
169	0,11	0,27	0,19	0,15
170	0,10	0,21	0,19	0,20
171	0,15	0,19	0,22	0,38
172	0,13	0,27	0,32	0,16
173	1,23	3,44	1,34	0,71
174	0,14	0,22	0,21	0,17
175	0,14	0,20	0,2	0,23
176	0,12	0,19	0,22	0,20
177	0,12	0,16	0,17	0,28
178	0,10	0,13	0,21	0,20
179	0,11	0,21	0,17	0,23

Fig 6

B

Entry site			Lymph node		
**WT**	**M45fs**	**M45fs-Rev**	**WT**	**M45fs**	**M45fs-Rev**
1,07E+07	5,53E+06	1,24E+07	2,98E+05	1,90E+05	4,49E+05
9,10E+06	2,07E+07	1,78E+07	3,39E+05	2,96E+05	7,00E+05
1,44E+07	8,28E+06	5,57E+06	6,37E+05	2,63E+05	6,44E+05
6,47E+07	1,17E+07	5,91E+06	7,95E+05	2,02E+05	3,21E+05
6,73E+06	6,59E+06	1,24E+07	7,62E+05	1,13E+05	5,63E+05

C

WT					M45fs			
**Cell No**	**IE1**	**m164**	**M105**	**m145**	**IE1**	**m164**	**M105**	**m145**
10000	4	11	5	7	5	12	9	7
10000	7	4	8	5	16	19	12	8
10000	3	8	6	4	4	10	10	7
25000	14	11	10	10	31	38	25	28
25000	15	18	12	9	24	33	14	14
25000	13	22	13	12	26	40	20	18
50000	35	29	16	19	28	58	43	43
50000	36	28	22	12	46	66	48	38
50000	35	30	20	20	45	64	49	46

D

	Gated in %	
**Library no**	**M45fs**	**M45fs-Rev**
2	0,32	0,26
1	0,30	0,31
3	0,24	0,19
4	0,28	0,27
5	0,38	0,25
6	0,49	0,33
7	0,38	0,26
8	0,30	0,27
9	0,33	0,15
10	0,27	0,23
11	0,23	0,25
12	0,31	0,19
13	0,26	0,23
14	0,38	0,35
15	0,37	0,28
16	0,38	0,27
17	0,31	0,18
18	0,41	0,30
19	0,33	0,29
20	0,37	0,35
21	0,36	0,36
22	0,34	0,26
23	0,21	0,23
24	0,35	0,34
25	0,16	0,33

Fig 6

26	0,43	0,28
27	0,24	0,26
28	1,32	0,55
29	0,31	0,26
30	0,35	0,36
31	0,27	0,32
32	0,33	0,33
33	0,41	0,29
34	0,33	0,22
35	0,37	0,22
36	0,32	0,27
37	0,27	0,19
38	0,28	0,14
39	0,32	0,13
40	0,30	0,26
41	0,34	0,25
42	0,34	0,30
43	0,28	0,31
44	0,34	0,20
45	0,39	0,20
46	0,27	0,21
47	0,36	0,31
48	0,34	0,22
49	0,24	0,13
50	0,24	0,18
51	0,30	0,29
52	0,22	0,26
53	0,34	0,29
54	0,31	0,24
55	0,28	0,22
56	0,31	0,22
57	0,37	0,25
58	0,38	0,29
59	0,34	0,22
60	0,31	0,16
61	0,26	0,27
62	0,35	0,25
63	0,33	0,29
181	0,31	0,13
64	0,30	0,20
66	0,36	0,26
67	0,34	0,22
68	0,36	0,26
69	0,33	0,24
70	0,30	0,22
71	0,26	0,32
72	0,26	0,24
73	0,30	0,13
74	0,38	0,17
75	0,26	0,28
76	0,23	0,30

Fig 6

77	0,36	0,29
78	0,31	0,28
79	0,83	0,37
80	0,22	0,26
81	0,35	0,26
82	0,33	0,39
83	0,40	0,29
84	0,29	0,32
85	0,22	0,24
86	0,26	0,20
87	0,30	0,19
88	0,35	0,15
89	0,31	0,16
90	0,24	0,24
91	0,28	0,27
92	0,27	0,26
93	0,30	0,17
94	1,17	0,54
95	0,43	0,17
96	0,30	0,18
97	0,29	0,30
98	0,16	0,35
99	0,33	0,23
101	0,34	0,19
102	0,83	0,33
103	0,35	0,24
104	0,27	0,23
105	0,77	0,30
106	0,35	0,23
107	0,35	0,20
108	0,35	0,20
110	0,29	0,30
111	0,32	0,19
112	0,21	0,13
113	0,30	0,28
114	0,35	0,23
115	0,28	0,25
116	0,30	0,20
117	0,30	0,21
118	0,56	0,30
119	0,28	0,20
120	0,23	0,27
121	0,31	0,20
122	0,27	0,18
123	0,34	0,28
124	0,30	0,22
125	0,32	0,20
126	0,24	0,24
127	0,29	0,22
129	1,28	0,55
133	0,24	0,27

Fig 6

134	0,34	0,21
135	0,25	0,24
136	0,29	0,14
137	0,37	0,34
138	0,27	0,23
139	0,33	0,25
140	0,22	0,19
141	0,32	0,25
142	0,35	0,21
143	0,26	0,16
144	0,28	0,13
145	0,37	0,22
146	0,31	0,19
147	0,31	0,25
148	0,27	0,22
149	0,41	0,25
150	0,30	0,19
151	0,30	0,18
152	0,36	0,24
153	0,37	0,19
154	1,66	0,40
155	0,25	0,23
156	0,22	0,19
157	0,34	0,20
158	0,24	0,20
159	0,33	0,16
160	0,25	0,23
161	0,38	0,19
162	0,23	0,22
163	0,27	0,15
164	0,36	0,21
165	0,22	0,18
166	0,28	0,23
167	0,35	0,28
168	0,31	0,21
169	0,27	0,16
170	0,27	0,24
171	0,35	0,23
172	0,30	0,13
173	2,98	0,83
174	0,23	0,22
175	0,32	0,13
176	0,22	0,15
177	0,34	0,13
178	0,27	0,14
179	0,30	0,20

Fig 7

C57BL/6

ΔM36

Cell No	M45	M57	m139	m141
10000	70	41	71	20
10000	67	36	54	28
10000	59	35	63	27
25000	136	73	134	37
25000	119	72	119	35
25000	112	76	137	36
50000	182	151	n.d.	75
50000	186	116	n.d.	63
50000	174	109	n.d.	50

ΔM36-Rev

Cell No	M45	M57	m139	m141
10000	41	23	41	16
10000	52	25	37	38
10000	44	24	39	21
25000	78	58	68	17
25000	68	50	73	36
25000	89	56	70	30
50000	127	86	n.d.	60
50000	121	88	n.d.	35
50000	121	91	n.d.	72

Unc93b1$^{3d/3d}$

ΔM36

Cell No	M45	M57	m139	m141
5000	68	29	36	25
5000	60	47	38	23
5000	55	33	32	35
10000	106	59	65	58
10000	111	71	56	57
10000	109	62	67	56
20000	189	104	101	94
20000	172	114	125	120
20000	207	123	128	126

ΔM36rev

Cell No	M45	M57	m139	m141
5000	84	30	81	18
5000	60	34	74	18
5000	68	19	78	22
10000	137	60	119	49
10000	121	52	116	32
10000	122	67	116	43
20000	191	115	174	72
20000	209	117	179	71
20000	216	119	174	77

Unc93b1$^{3d/3d}$

ΔM36

Cell No	M45	M57	m139	m141
5000	63	39	38	21
5000	53	34	45	23
5000	59	38	41	22
10000	86	62	53	33
10000	91	51	75	26
10000	82	64	74	45
20000	156	99	101	58
20000	154	99	111	55
20000	139	101	121	57

ΔM36-FADD

Cell No	M45	M57	m139	m141
5000	78	37	53	32
5000	60	43	50	21
5000	62	40	66	26
10000	98	57	79	40
10000	97	68	108	38
10000	87	57	105	48
20000	173	72	135	67
20000	156	117	122	69
20000	156	91	126	60

Clodronate Liposomes

Cell No	IE1	m164	M105	m145
5000	2	2	1	4
5000	1	1	3	5
5000	5	1	0	0
10000	4	2	2	5
10000	3	3	0	1
10000	6	2	0	4
20000	3	3	1	8
20000	1	3	3	8
20000	4	3	1	3

PBS Liposomes

Cell No	IE1	m164	M105	m145
5000	1	4	0	6
5000	4	9	1	3
5000	0	3	0	0
10000	4	8	2	3
10000	10	4	1	2
10000	4	7	1	5
20000	9	4	4	9
20000	12	15	1	10
20000	12	14	1	12

S2 Fig

A **day 2**

ΔM36

Cell No	M45	M57	m139	m141
10000	0	0	1	1
10000	0	0	1	2
10000	0	2	4	2
25000	3	6	3	4
25000	0	5	6	5
25000	6	3	7	3
50000	4	10	12	11
50000	6	11	8	6
50000	7	8	8	7

ΔM36-Rev

Cell No	M45	M57	m139	m141
10000	1	3	0	1
10000	1	2	0	2
10000	3	4	2	3
25000	4	0	5	6
25000	7	3	2	4
25000	4	2	2	3
50000	7	9	8	8
50000	5	4	7	8
50000	7	6	7	8

day 3

ΔM36

Cell No	M45	M57	m139	m141
10000	0	2	0	0
10000	0	0	2	0
10000	0	2	2	1
25000	2	2	8	3
25000	3	4	4	3
25000	5	0	7	2
50000	17	3	4	5
50000	26	6	7	2
50000	18	9	11	6

ΔM36-Rev

Cell No	M45	M57	m139	m141
10000	0	0	1	1
10000	2	1	1	0
10000	1	2	2	1
25000	8	5	3	1
25000	6	3	2	1
25000	5	2	4	2
50000	10	6	2	0
50000	10	9	3	0
50000	26	2	7	4

day 4

ΔM36

Cell No	M45	M57	m139	m141

ΔM36-Rev

Cell No	M45	M57	m139	m141

10000	3	2	1	0
10000	6	0	0	0
10000	1	3	0	3
25000	14	5	7	3
25000	10	5	2	4
25000	14	1	2	3
50000	13	7	9	0
50000	9	10	7	8
50000	19	7	7	6

10000	5	0	3	0
10000	4	2	3	5
10000	2	2	1	0
25000	12	3	5	2
25000	7	5	3	5
25000	12	3	4	5
50000	17	13	8	4
50000	14	14	11	10
50000	23	9	8	8

day 5

ΔM36

Cell No	M45	M57	m139	m141
10000	6	5	3	4
10000	3	4	7	4
10000	9	0	8	7
25000	19	22	17	11
25000	23	25	24	12
25000	16	16	15	16
50000	32	x	x	19
50000	29	x	x	23
50000	40	x	x	19

ΔM36-Rev

Cell No	M45	M57	m139	m141
10000	4	1	6	6
10000	1	4	2	4
10000	2	5	1	1
25000	13	11	9	6
25000	12	14	5	7
25000	14	13	7	5
50000	27	26	10	11
50000	25	14	10	21
50000	29	18	14	8

day 6

ΔM36

Cell No	M45	M57	m139	m141
10000	12	4	4	4
10000	10	6	4	3
10000	9	6	3	3

ΔM36-Rev

Cell No	M45	M57	m139	m141
10000	5	8	2	7
10000	5	6	2	6
10000	7	5	3	3

25000	22	25	20	13
25000	23	19	15	7
25000	21	18	13	13
50000	44	28	27	25
50000	40	35	30	18
50000	35	32	23	20

25000	15	12	7	7
25000	20	11	10	9
25000	15	15	11	9
50000	45	20	23	19
50000	39	18	25	18
50000	33	13	14	21

day 7

ΔM36

Cell No	M45	M57	m139	m141
10000	11	7	8	9
10000	9	7	12	9
10000	10	10	11	15
25000	32	26	21	27
25000	26	26	32	30
25000	28	30	35	30
50000	52	54	52	53
50000	53	43	51	57
50000	45	48	45	46

ΔM36-Rev

Cell No	M45	M57	m139	m141
10000	7	5	7	9
10000	13	4	9	6
10000	8	13	10	8
25000	21	17	14	21
25000	22	28	12	23
25000	22	18	17	20
50000	42	34	33	30
50000	45	37	34	39
50000	44	22	32	32

B Spleen

day 7

ΔM36

Cell No	IE1	m164	M105	m145
5000	34	88	29	55
5000	39	93	38	51
5000	47	88	34	46

ΔM36-Rev

Cell No	IE1	m164	M105	m145
5000	52	75	20	41
5000	43	65	25	28
5000	46	88	33	48

10000	66	164	54	88
10000	56	157	46	101
10000	64	193	72	86
20000	122	236	127	152
20000	115	240	95	151
20000	129	275	102	150

10000	72	129	55	66
10000	99	136	51	65
10000	105	119	42	57
20000	140	188	78	119
20000	141	195	67	120
20000	142	207	68	116

3 months

MZ ΔM36

Cell No	IE1	m164	M105	m145
10000	42	50	11	22
10000	42	46	13	12
10000	39	49	9	20
25000	106	102	23	41
25000	96	114	29	50
25000	91	126	23	49
50000	171	210	51	88
50000	157	181	62	87
50000	173	235	43	81

ΔM36-Rev

Cell No	IE1	m164	M105	m145
10000	42	36	7	22
10000	41	33	8	11
10000	34	35	11	9
25000	74	78	14	35
25000	83	82	15	34
25000	95	71	17	24
50000	170	157	37	52
50000	179	184	41	62
50000	185	178	35	56

21 months

ΔM36

Cell No	IE1	m164	M105	m145
10000	20	35	16	8
10000	30	36	22	17
10000	19	27	13	16

ΔM36-Rev

Cell No	IE1	m164	M105	m145
10000	59	70	15	18
10000	54	70	14	25
10000	69	75	16	24

S2 Fig

25000	41	69	28	26
25000	50	81	22	31
25000	43	62	42	27
50000	82	135	62	46
50000	75	123	67	54
50000	96	136	69	59

25000	141	154	38	68
25000	152	149	22	83
25000	130	148	50	69
50000	211	232	56	121
50000	n.d.	n.d.	76	117
50000	n.d.	n.d.	51	117

S3 Fig

A

entry site		Lymph node	
**WT.BAC**	**ΔM27**	**WT.BAC**	**ΔM27**
1,00E+07	4,41E+06	9,77E+05	3,30E+05
2,62E+07	8,20E+06	1,15E+06	4,02E+05
1,46E+07	3,14E+07	1,45E+06	1,10E+06
7,78E+06	8,26E+06	6,42E+05	5,96E+05
1,29E+07	7,40E+06	1,35E+06	5,66E+05
1,18E+07	8,62E+06	8,25E+05	2,65E+05
1,77E+07	1,74E+07	8,45E+05	4,88E+05

B

WT.BAC

**Cell No**	IE1	m164	M105	m145
10000	101	65	43	95
10000	122	83	57	87
10000	141	81	63	71
25000	241	201	125	133
25000	243	188	154	156
25000	249	227	120	168

ΔM27

**Cell No**	IE1	m164	M105	m145
10000	38	72	15	59
10000	40	59	14	45
10000	44	50	27	45
25000	87	103	69	109
25000	100	136	58	76
25000	105	116	60	103
50000	191	211	75	149
50000	190	150	85	147
50000	186	195	93	152
